# ICU Readmission and In-Hospital Mortality Rates for Patients Discharged from the ICU—Risk Factors and Validation of a New Predictive Model: The Worse Outcome Score (WOScore)

**DOI:** 10.3390/jpm15100479

**Published:** 2025-10-03

**Authors:** Eleftherios Papadakis, Athanasia Proklou, Sofia Kokkini, Ioanna Papakitsou, Ioannis Konstantinou, Aggeliki Konstantinidi, Georgios Prinianakis, Stergios Intzes, Marianthi Symeonidou, Eumorfia Kondili

**Affiliations:** 1 Department of Intensive Care Medicine, University Hospital of Heraklion, 71110 Heraklion, Greece; medp2011920@med.uoc.gr (E.P.); aproklou@pagni.gr (A.P.); sofkok@hotmail.com (S.K.); medp2011875@med.uoc.gr (I.P.); konstantinou.ioannis@yahoo.gr (I.K.); akonstantinidi@pagni.gr (A.K.); gprinianakis@pagni.gr (G.P.); 2 Medical School, University of Crete, 70013 Heraklion, Crete, Greece; 3 Department of Management Science and Technology, Democritus University, 65404 Kavala, Greece; instergios@hotmail.com; 4 Department of Hematology, Medical School, Democritus University of Thrace, Area of Dragana, 68100 Alexandroupolis, Greece; marianthi.symeonidou@hotmail.com

**Keywords:** readmission, in-hospital mortality, Intensive Care Unit (ICU)

## Abstract

**Background**: Intensive Care Unit (ICU) readmission and in-hospital mortality are critical indicators of patient outcomes following ICU discharge. Patients readmitted to the ICU often face worse prognosis, higher healthcare costs, and prolonged hospital stays. Identifying high-risk patients is essential for optimizing post-ICU care and resource allocation. **Methods**: This two-phase study included the following: (1) a retrospective analysis of ICU survivors in a mixed medical–surgical ICU to identify risk factors associated with ICU readmission and in-hospital mortality, and (2) a prospective validation of a newly developed predictive model: the Worse Outcome Score (WOScore). Data collected included demographics, ICU admission characteristics, severity scores (SAPS II, SAPS III, APACHE II, SOFA), interventions, complications and discharge parameters. **Results**: Among 1.190 ICU survivors, 126 (10.6%) were readmitted to the ICU, and 192 (16.1%) died in hospital after ICU discharge. Key risk factors for ICU readmission included Diabetes Mellitus, SAPS III on admission, and ICU-acquired infections (Ventilator-Associated Pneumonia (VAP) and Catheter-Related Bloodstream Infection, (CRBSI)). Predictors of in-hospital mortality were identified: medical admission, high SAPS III score, high lactate level on ICU admission, tracheostomy, reduced GCS at discharge, blood transfusion, CRBSI, and Acute Kidney Injury (AKI) during ICU stay. The WOScore, developed based on the results above, demonstrated strong predictive ability (AUC: 0.845 derivation, 0.886 validation). A cut-off of 20 distinguished high-risk patients (sensitivity: 88.1%, specificity: 73.0%). **Conclusions**: ICU readmission and in-hospital mortality are influenced by patient severity, underlying comorbidities, and ICU-related complications. The WOScore provides an effective, easy-to-use risk stratification tool that can guide clinicians in identifying high-risk patients at ICU discharge and guide post-ICU interventions, potentially improving patients’ outcomes and optimizing resource allocation. Further multi-center studies are necessary to validate the model in diverse healthcare settings.

## 1. Background

In-hospital mortality and ICU readmission after discharge are among the most significant adverse outcomes for patients admitted to Intensive Care Units (ICUs). Reported in-hospital mortality and ICU readmission rates after ICU discharge vary considerably across studies, reflecting differences in patient populations, case mix, and healthcare settings [1,2,3]. ICU readmitted patients typically have poorer prognoses, including higher mortality rates and prolonged hospital stays [4,5]. Furthermore, deaths occurring after prolonged ICU stays contribute to increased healthcare costs without achieving the desired clinical benefits, thus straining healthcare resources [6,7].

Factors contributing to ICU readmission and in-hospital mortality after ICU discharge are multifaceted, including patient-specific characteristics, clinical conditions on ICU admission and discharge, complications and interventions during ICU hospitalization, and organizational factors of patient management [4,8,9,10,11,12]. Due to this complex etiology, using these rates as direct quality indicators for hospital comparisons is challenging [1,13].

Although widely used in critical care, existing severity scoring systems such as APACHE, SOFA, and SAPS were originally developed to estimate in-ICU mortality and are not optimized for predicting adverse outcomes after ICU discharge. Their predictive performance for post-ICU mortality or readmission is limited, with area under the curve (AUC) values often below 0.80 in contemporary validation studies [14]. These scores do not account for post-discharge physiological instability, functional status, or ICU-acquired complications. The COVID-19 pandemic has further highlighted this gap: critically ill survivors, particularly those who develop complications such as VAP, face heightened risks of deterioration and readmission [15,16]. Given these limitations, there remains a clear need for a pragmatic, accurate, and externally validated predictive model tailored to heterogeneous post-ICU populations.

Targeted measures remain essential to reduce adverse post-ICU outcomes. Implementing standardized protocols for ICU admission and discharge, along with structured transition programs, can facilitate smoother transfers to general hospital wards [17]. Such programs may involve specialized ICU outreach teams or ICU liaison nurses, ensuring consistent follow-up care and early identification of deterioration in discharged patients [18,19]. While transition programs have shown promise in improving post-ICU outcomes, their implementation entails significant costs [20,21,22]. Therefore, identifying high-risk patients is crucial to optimizing resource allocation. Developing and validating predictive models for adverse outcomes after ICU discharge are critical steps towards improving patient care [23,24,25].

The primary objectives of this study are as follows: (1) to identify and analyze the key risk factors associated with ICU readmission, (2) to determine the risk factors contributing to in-hospital mortality among patients discharged from the ICU, (3) to evaluate the risk factors for worse outcomes, including readmission or in-hospital death, in this patient population, (4) to develop a robust predictive model capable of identifying patients at higher risk for worse outcomes after ICU discharge, and (5) to prospectively validate the predictive model to ensure its accuracy, and reliability.

## 2. Methods

### 2.1. Study Design

The present study consists of two phases: (a) a single-center retrospective observational study aimed at identifying risk factors for ICU readmission and in-hospital mortality and developing a predictive model to detect high-risk patients for worse outcomes after ICU discharge; and (b) a prospective validation of the predictive model developed in the first phase. Both phases were conducted in a mixed (medical–surgical) University Intensive Care Unit with a capacity of 12 beds. The ICU operates as a closed unit with the continuous presence of specialized medical staff without strict protocols governing patient admission and discharge.

#### 2.1.1. Outcome Definitions

Initial ICU admission was defined as the patient’s first admission to the ICU during a single hospitalization. ICU readmission was defined as the first readmission occurring within seven days after ICU discharge during the same hospitalization. In-hospital mortality was defined as death occurring after ICU discharge but before hospital discharge.

The composite endpoint, “worse outcome,” was defined as either ICU readmission or in-hospital mortality.

#### 2.1.2. Ethical Considerations

The study was conducted in accordance with the Declaration of Helsinki and approved by the Research Ethics Committee of the University Hospital of Heraklion (Approval Code: 12, Approval Date: 8 January 2020), following a recommendation from the hospital’s Scientific Council (Approval Code: 11222, Date: 13 November 2019).

Informed consent was not required because the study used existing records or data and did not involve an interventional procedure nor affect clinical management. The study posed minimal risk, ensured confidentiality, and protected participant privacy and dignity.

### 2.2. Population and Data Collection

All patients admitted to the Intensive Care Unit of the University General Hospital of Heraklion between 1 January 2015 and 14 March 2020 were considered.

#### 2.2.1. Inclusion Criteria

Patients > 16 years old who were admitted during the period 1 January 2016–14 March 2020 in the ICU and were discharged to other general wards of the hospital.

#### 2.2.2. Exclusion Criteria

Patients who died during their first admission in the ICU.

Patients discharged for palliative care in a ward.

Patients who were transferred to another hospital.

Patients with ICU hospitalization less than 24 h.

Data were retrieved from hospital electronic records and anonymized prior to analysis. All outcomes which were extracted from the hospital’s electronic medical records were verified by two clinicians during data extraction to ensure accuracy and reliability and no external adjudication process was applied.

### 2.3. Variable Selection

Variables collected encompassed patient demographics, comorbidities, clinical scores, interventions, complications during ICU stay, and discharge parameters.

A detailed list of all variables examined is provided in Supplementary Materials (Table S2).

Variables were categorized as pre-specified based on clinical relevance and literature review, while exploratory variables were tested during analysis. Only statistically significant and clinically meaningful variables were retained for model development.

### 2.4. Statistical Modeling

All statistical analyses were performed using IBM SPSS Statistics, version 29 (IBM Corp., Armonk, NY, USA). For analyses not supported by SPSS, including calculation of Brier scores and Decision Curve Analysis (DCA), additional software such as R (version 4.4.2; R Foundation for Statistical Computing, Vienna, Austria) or Python (version 3.12.6; Python Software Foundation, Wilmington, DE, USA) was used.

Categorical variables are presented as frequencies and percentages, and continuous variables are expressed as means ± standard deviation (SD) for normally distributed data or medians with interquartile ranges (IQR) for non-normally distributed data. The Kolmogorov–Smirnov and Shapiro–Wilk tests were used to assess the normality of distributions. Categorical variables were compared using the chi-square test or Fisher’s exact test as appropriate, while continuous variables were compared using the independent samples *t*-test or Mann–Whitney U test, depending on data distribution. Missing data were assessed for all variables. Variables with >10% missingness were excluded from the analyses, while variables with low missingness were imputed using the median.

Variables entered into the statistical models were pre-specified based on clinical relevance and previous literature, as described in Section 2.3. Univariate logistic regression analyses were conducted to assess the association between potential risk factors and the primary outcomes: ICU readmission, in-hospital mortality, and the composite endpoint (worse outcome). Prior to multivariate modeling, all severity scores assessed at ICU admission and discharge (SAPS II, SAPS III, APACHE II, APACHE IV, SOFA, qSOFA) were evaluated for discriminative ability using ROC analysis. For each time point, only the score with the highest AUC was selected for inclusion to avoid collinearity among severity scores. Multivariate logistic regression was then performed using a stepwise backward elimination approach, guided by statistical significance (*p* < 0.05) and clinical relevance. Multicollinearity was assessed using variance inflation factors (VIF), with variables exceeding a VIF of 5 excluded from the final models. Adjusted odds ratios (ORs) with 95% confidence intervals (CIs) were reported.

Model discrimination was assessed using receiver operating characteristic (ROC) curves and area under the curve (AUC) with 95% CIs. Calibration was evaluated in the derivation cohort using the Hosmer–Lemeshow goodness-of-fit test and calibration plots, and overall accuracy was assessed with the Brier score and Nagelkerke’s R^2^. No internal validation (e.g., bootstrap resampling or cross-validation) was performed, as the model was developed in the retrospective derivation cohort with a priori prospective external validation in an independent sample. Clinical utility was evaluated with DCA, comparing the net benefit of the WOScore against established severity scores. Sensitivity analyses were performed for alternative cut-off values to assess robustness. Baseline characteristics of derivation and validation cohorts were compared to assess transportability.

### 2.5. Score Construction and Validation

The WOScore was developed using independent predictors identified in the multivariate logistic regression model, selected based on statistical significance, absence of multicollinearity, and clinical relevance. Point values for each variable were assigned proportionally to their adjusted ORs from the final model. Beta coefficients were reviewed to ensure consistency with the OR-based weighting, and point allocations were rounded to the nearest integer to facilitate clinical application. The total WOScore was calculated as the sum of these points.

The optimal cut-off for high-risk classification was determined in the derivation cohort using Youden’s index to balance sensitivity and specificity. This cut-off (≥20 points) was applied without recalibration in the prospective validation phase. Sensitivity analyses were conducted using thresholds of 15 and 25 points to confirm stability of performance metrics.

Prospective external validation was performed in an independent cohort of ICU survivors discharged to hospital wards between 1 January and 30 April 2023, excluding patients discharged for palliative care or transferred to other hospitals, calculating the score at patients’ discharge. The WOScore was applied without recalibration (as originally derived)**,** and validation analyses used the same discrimination, calibration, and clinical utility metrics as in the derivation phase. WOScore performance was compared with the most predictive conventional severity scores identified—SAPS III at ICU admission and SAPS II at ICU discharge—using AUC, Brier score, Hosmer–Lemeshow test, and Decision Curve Analysis.

## 3. Results

During the retrospective derivation phase (1 January 2015–14 March 2020), 1877 patients were admitted to the ICU. Of these, 401 (21.4%) died during their ICU stay and were not eligible for further analysis. Among the 1476 ICU survivors, 286 patients were excluded: 249 due to ICU stays shorter than 24 h, 33 because they were transferred to another hospital, and 4 who were discharged for palliative care. The final derivation cohort therefore comprised 1190 patients.

**Figure 1:**
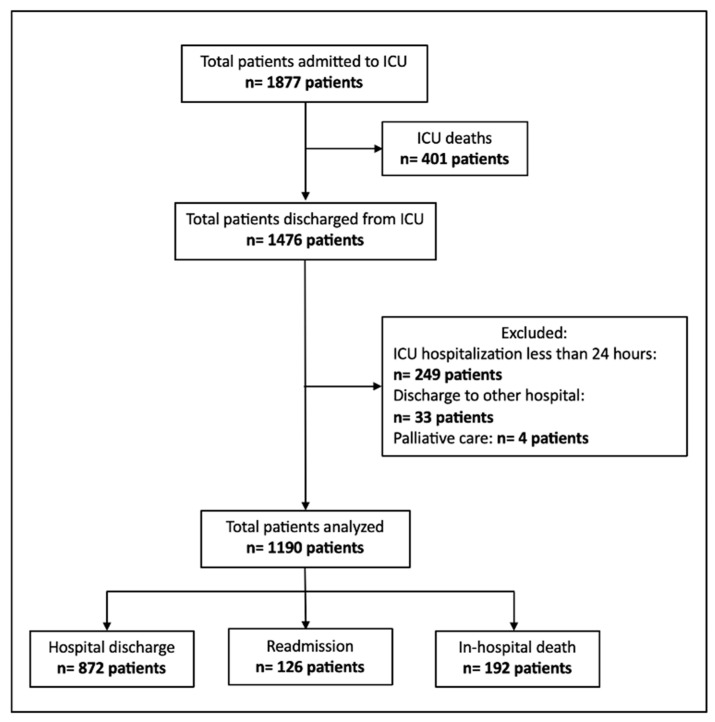
Flowchart of patient selection for the retrospective derivation cohort, showing inclusion, exclusion, and final patient outcomes after ICU discharge.

Of these, 872 patients (73.3%) were discharged from the hospital without ICU readmission, 126 patients (10.6%) were readmitted to the ICU during the same hospitalization, and 192 patients (16.1%) died on the ward after ICU discharge (Figure 1).

### 3.1. Risk Factors for ICU Readmission

**Figure 2:**
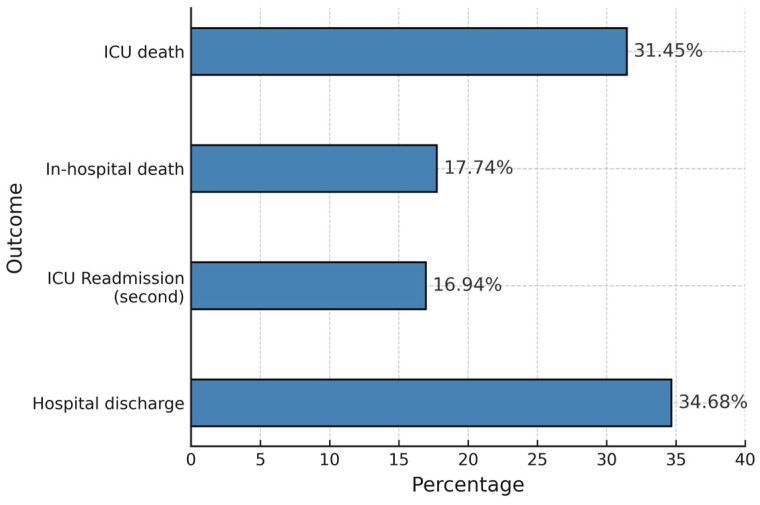
Outcomes of readmitted ICU patients. The bar chart illustrates the distribution of post-readmission outcomes.

During the retrospective derivation phase, 126 (10.6%) of the 1190 analyzed ICU survivors were readmitted to the ICU at a mean interval of 3.22 days (SD 1.86) after ICU discharge. The mean ICU length of stay for these readmissions was 12.63 days (SD 20.18). The most common causes of ICU readmission were septic shock and acute respiratory failure (with or without ARDS) accounted for more than 70% of cases (Supplementary Materials: Figure S1). As illustrated in Figure 2, outcomes after ICU readmission were poor: Nearly half of the readmitted patients (49.19%) did not survive hospitalization, with a significant proportion dying in the ICU or after ICU discharge. Only about one-third of readmitted patients were successfully discharged (34.68%), while a notable percentage required a second ICU readmission (16.94%).

In multivariate logistic regression analysis, five variables were independently associated with ICU readmission (Table 1). Diabetes Mellitus increased the odds of readmission by 57% (OR = 1.569, 95% CI: 1.182–2.507, *p* = 0.047). SAPS III score on ICU admission was significantly associated with ICU readmission, with each additional point increasing the odds by 2.2% (OR = 1.022, 95% CI: 1.002–1.042, *p* = 0.029). Among complications during the ICU stay, VAP was significantly associated with higher odds of ICU readmission (OR = 1.749, 95% CI: 1.040–2.943, *p* = 0.035). The strongest association was observed with CRBSI, which increased the likelihood of ICU readmission by 2.5 times (OR = 2.520, 95% CI: 1.494–4.251, *p* < 0.001).

From all severity scores tested at ICU admission and discharge, only the score with the highest discriminative ability (based on AUC) at each time point was included in the multivariate model to avoid collinearity. Specifically, SAPS III on ICU admission (AUC = 0.639) and APACHE II at ICU discharge (AUC = 0.611) were selected as the most predictive scores for readmission (Supplementary Materials: Figure S2). In the Supplemental Material, Table S3 presents the detailed descriptive statistical results of patients who were readmitted to the ICU, and Table S4 presents the complete results of the univariate and multivariate analyses.

### 3.2. Risk Factors for In-Hospital Mortality

Among ICU survivors discharged to the ward in derivation cohort, a total of 192 patients (16.1%) died during the same hospitalization. Compared with survivors, non-survivors had a higher burden of acute illness at admission, more frequent ICU-acquired complications, and worse physiological status at discharge.

In the multivariate logistic regression analysis (Table S4), several factors were identified as independent predictors of in-hospital mortality after ICU discharge. Medical admission was associated with more than a twofold increase in mortality risk (OR = 2.247, 95% CI: 1.294–3.904, *p* = 0.004). Elevated lactate levels on ICU admission (OR = 1.014, 95% CI: 1.005–1.023, *p* = 0.003) and failure to achieve lactate clearance at 48 h (OR = 1.750, 95% CI: 1.070–2.864, *p* = 0.026) were also significant predictors. The presence of AKI (OR = 1.671, 95% CI: 1.005–2.777, *p* = 0.048) and receipt of blood products transfusion (OR = 2.240, 95% CI: 1.360–3.690, *p* = 0.002) independently increased mortality risk. Among ICU-acquired infections, CRBSI (OR = 1.947, 95% CI: 1.103–3.437, *p* = 0.021) was also independently associated with in-hospital mortality. Procedural and neurological parameters were also strongly linked to outcome: Tracheostomy at ICU discharge was the strongest predictor, with a nearly fourfold increase in the odds of in-hospital death (OR = 3.956, 95% CI: 2.275–6.879, *p* < 0.001). Finally, reduced GCS (OR = 0.882, 95% CI: 0.817–0.951, *p* = 0.001) contributes to the increase in in-hospital mortality (Table 2).

Regarding severity scores, only those with the highest discriminative ability were included in the final multivariate analysis. Specifically, SAPS III on ICU admission (AUC (0.811, 95% CI: 0.782–0.841) and SAPS II (AUC = 0.823, 95% CI: 0.793–0.853) on ICU discharge outperformed other scores assessed (Supplementary Materials: Figure S3). Of these, only SAPS III on patients’ admission was identified as an independent prognostic factor (OR = 1.046, 95% CI: 1.024–1.069, *p* = <0.001).

These findings indicate that in-hospital mortality after ICU discharge is influenced by a combination of admission severity, failure to resolve early metabolic derangements, ICU-acquired complications, and poor physiological reserve at discharge.

The complete set of descriptive statistics, univariate, and multivariate analysis results for in-hospital mortality in the derivation cohort are provided in the Supplementary Materials (Tables S5 and S6).

### 3.3. Risk Factors for Worse Outcome (ICU Readmission or In-Hospital Mortality)

**Figure 3:**
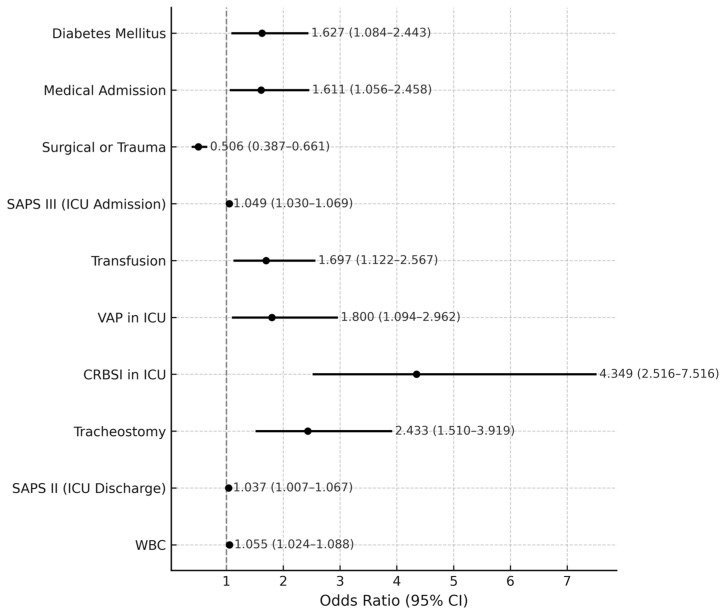
Forest plot depicting the adjusted odds ratios (OR) and 95% confidence intervals (CI) for predictors of worse outcomes after ICU discharge. The red dashed line represents the reference line at OR = 1.0. Variables with OR > 1.0 indicate an increased risk of worse outcomes, while those with OR < 1.0 suggest a protective effect. Error bars indicate 95% confidence intervals.

**Figure 4:**
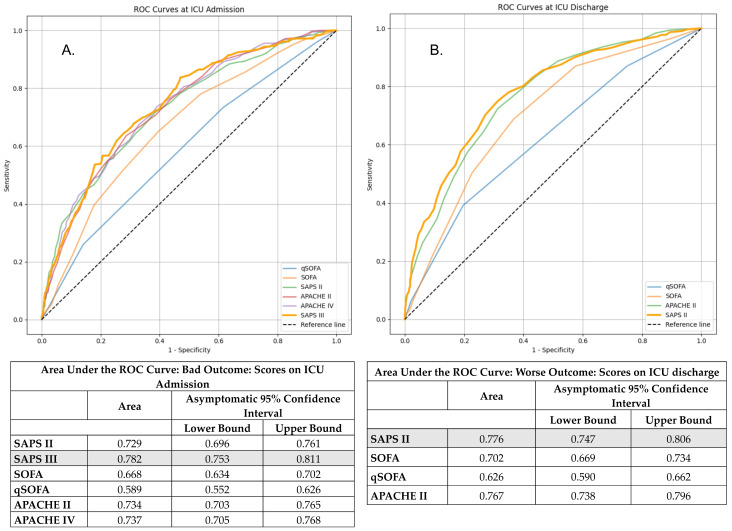
Receiver operating characteristic (ROC) curves for predicting worse outcome (ICU readmission or in-hospital death) using clinical severity scores at ICU admission (**A**) and ICU discharge (**B**).

Worse outcome was defined as the composite endpoint including either ICU readmission or in-hospital death after ICU discharge. In the derivation cohort, 318 of 1190 patients (26.7%) experienced this composite endpoint. The complete descriptive statistics are provided in Table S7 in Supplements. The results of the univariate and multivariate logistic regression analyses are summarized in Table 3, while a graphical representation of independent predictors is displayed in the forest plot (Figure 3). Comparative performance of severity scores is presented in Figure 4.

In the multivariate logistic regression analysis, several factors were identified as independent predictors of worse outcome. Among patient characteristics, Diabetes Mellitus remained a significant risk factor (OR = 1.627, 95% CI: 1.084–2.443, *p* = 0.019), while medical admission was also associated with an increased likelihood of worse outcome (OR = 1.611, 95% CI: 1.056–2.458, *p* = 0.027).

Most clinical severity scores, both on ICU admission and during ICU discharge, showed a statistically significant difference between the two outcome groups in the univariate analysis. Based on AUC results, SAPS III on ICU admission (AUC: 0.782) and SAPS II on ICU discharge (AUC: 0.776) were included in the multivariate model due to their superior discriminative ability compared to alternative scores (Figure 3). In the multivariate analysis, both SAPS III (admission) (OR = 1.049, 95% CI: 1.030–1.069, *p* < 0.001) and SAPS II (discharge) (OR = 1.037, 95% CI: 1.007–1.067, *p* = 0.014) remained statistically significant predictors of worse outcome (Table 3).

At ICU discharge, tracheostomy emerged as the strongest independent predictor, nearly tripling the odds of worse outcome (OR = 2.433, 95% CI: 1.510–3.919, *p* < 0.001)., while higher WBC count also conferred increased risk (OR = 1.055, 95% CI: 1.024–1.088, *p* < 0.001).

Among ICU-related complications: VAP was independently associated with worse outcome (OR = 1.800, 95% CI: 1.094–2.962, *p* = 0.021), and CRBSI showed the highest impact, more than quadrupling the risk (OR = 4.349, 95% CI: 2.516—7.516, *p* < 0.001). Transfusion of blood products also remained a statistically significant factor (OR = 1.697, 95% CI: 1.122–2.567, *p* = 0.012) (Figure 3).

### 3.4. Model Construction (Derivation Cohort)

To predict the risk of ICU readmission or in-hospital mortality after ICU discharge, we developed the Worse Outcome Score (WOS) based on the findings of the previously described multivariate logistic regression analysis with stepwise backward elimination. The score incorporates eight independent risk factors, all of which were significantly associated with adverse outcomes. Point values were assigned to each variable proportionally to their adjusted odds ratios, with confirmation against beta coefficients to preserve consistency (Table 4). The final WOScore ranged from –5 to 75 points, with higher scores indicating greater risk of adverse outcomes.

#### 3.4.1. Comparison of WOS by Outcome Groups in the Derivation Cohort

Patients who experienced the composite endpoint of ICU readmission or in-hospital death had significantly higher WOScore values compared to those discharged alive without adverse events (38.55 ± 17.9 vs. 15.67 ± 14.7, *p* < 0.001). This difference remained consistent across the derivation cohort, confirming the ability of the score to distinguish high- from low-risk groups. Each one-point increase in WOScore was associated with an 8% increase in the odds of a worse outcome (OR: 1.082, 95% CI: 1.072–1.093). Detailed results are shown in Table 5.

#### 3.4.2. Cut-Off Validation and Classification Performance

A cut-off score of 20 was selected as the optimal threshold for risk stratification using Youden’s index. At this cut-off, the WOScore achieved a sensitivity of 86.8% and specificity of 61.1%. The positive predictive value (PPV) was 44.9%, indicating that nearly half of patients classified as high risk experienced a worse outcome, while the negative predictive value (NPV) was 92.7%, confirming the ability of the WOScore to reliably identify patients at low risk. To further assess the robustness of this threshold, we performed a sensitivity analysis exploring alternative cut-offs of 15 and 25 points, confirming the choice of 20 as the optimal cut-off for clinical application. Classification results are summarized in Table 6.

#### 3.4.3. Predictive Performance of the WOS

**Figure 5:**
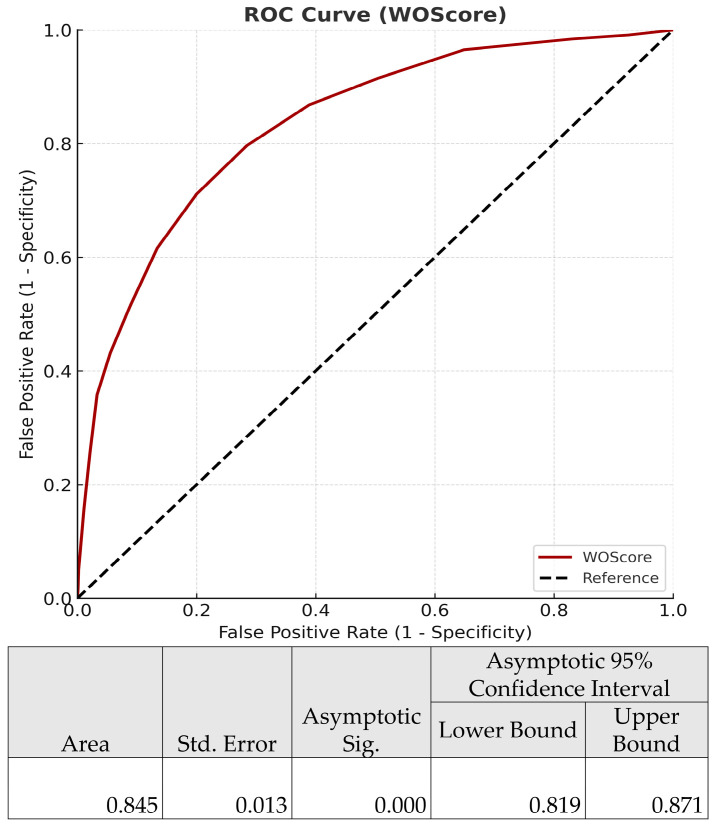
Receiver operating characteristic (ROC) curve for the Worse Outcome Score (WOS) in predicting ICU readmission or in-hospital mortality.

The WOS ≥ 20 threshold was strongly associated with an increased risk of worse outcome, with an Odds Ratio of 10.313 (95% CI: 7.252–14.664, *p* < 0.001). The AUC for WOS was 0.845 (95% CI: 0.819–0.871, *p* < 0.001), demonstrating excellent discriminative ability (Figure 5).

**Figure 6:**
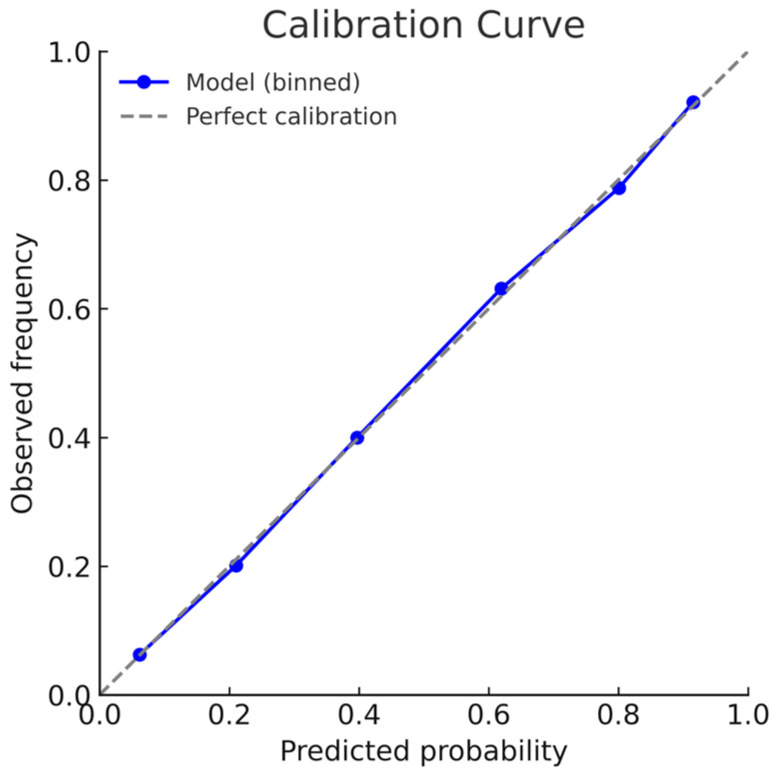
Calibration curve of the predictive model. Observed event frequencies are plotted against predicted probabilities in deciles of risk (blue line and markers). The dashed gray line represents perfect calibration. The close agreement of the model curve with the reference line indicates excellent calibration across the risk spectrum.

Model calibration was also very good: the Hosmer–Lemeshow test indicated no evidence of misfit (*p* = 0.721), while the calibration intercept (~0.00002) and slope (~1.00003) closely approximated ideal values. The calibration curve (Figure 6) illustrates the close agreement between predicted and observed event probabilities across the full range of risk. Overall accuracy was supported by a Brier score of 0.128, a Brier Skill Score of 0.34 indicating added value over baseline prediction, while the Nagelkerke R^2^ value of 0.51 indicated that the model explained more than half of the variance in outcomes, underscoring its strong predictive ability.

Decision Curve Analysis (DCA) further confirmed the clinical utility of the WOScore. Compared with strategies of treating all or treating none, use of the WOScore provided a consistently positive net benefit across a wide range of threshold probabilities (0.1–0.6). For example, at a threshold of 0.2, application of the WOScore correctly identified approximately 17 additional patients per 100 at risk of worse outcomes, after accounting for false positives. Net benefit values at thresholds of 0.1, 0.2, 0.3, 0.4, and 0.5 were 0.207, 0.168, 0.142, 0.107, and 0.088, respectively (in Supplementary Materials Table S8). These findings support the use of the WOScore as a pragmatic tool for guiding post-ICU decision-making.

### 3.5. Model Validation

#### 3.5.1. Prospective Validation of the Worse Outcome Score (WOS)

**Figure 7:**
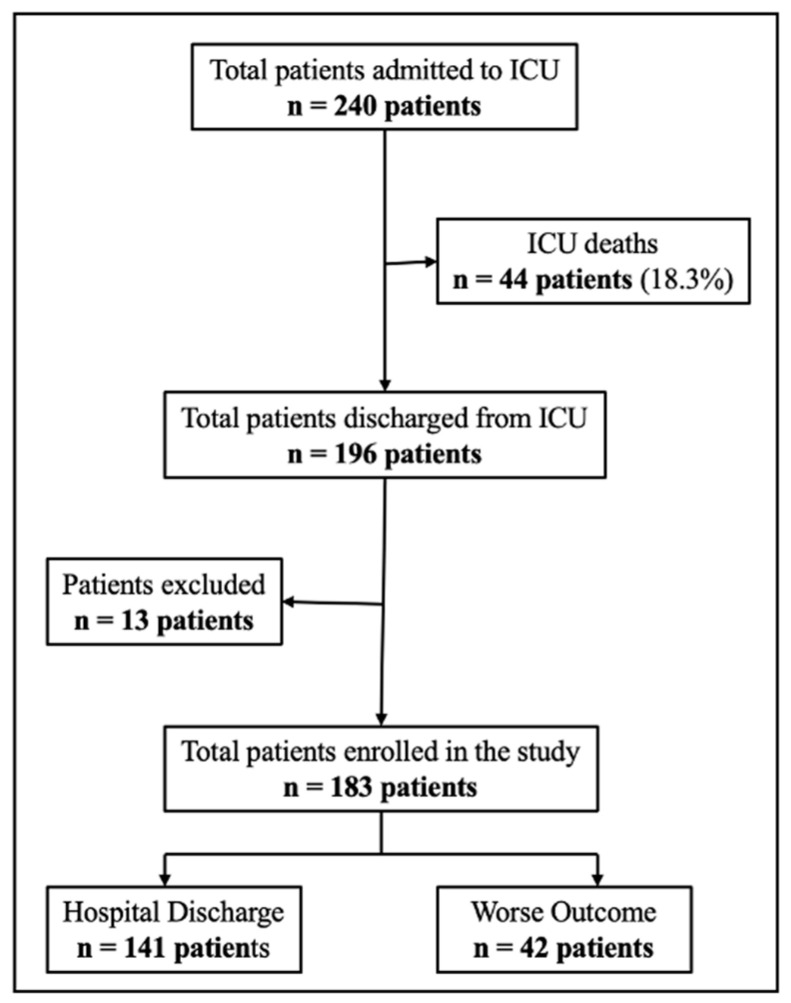
Flowchart of patient inclusion in the WOScore validation trial.

To evaluate the external performance and clinical applicability of the Worse Outcome Score (WOS), we conducted a prospective validation in ICU survivors discharged between January 2023–April 2023. Of the 240 patients admitted to the ICU, 44 (18.3%) died before ICU discharge. Among the 196 ICU survivors, 13 did not meet inclusion criteria and 183 patients met the inclusion criteria and were followed until hospital discharge. (Figure 7).

A comparison of baseline characteristics between the derivation (n = 1190) and validation cohort (n = 183) demonstrated no significant differences in demographic, clinical, outcomes or severity indices, supporting the transportability of the WOS (Supplementary Materials Table S9).

#### 3.5.2. Comparison of WOS by Outcome Groups in the Validation Cohort

**Figure 8:**
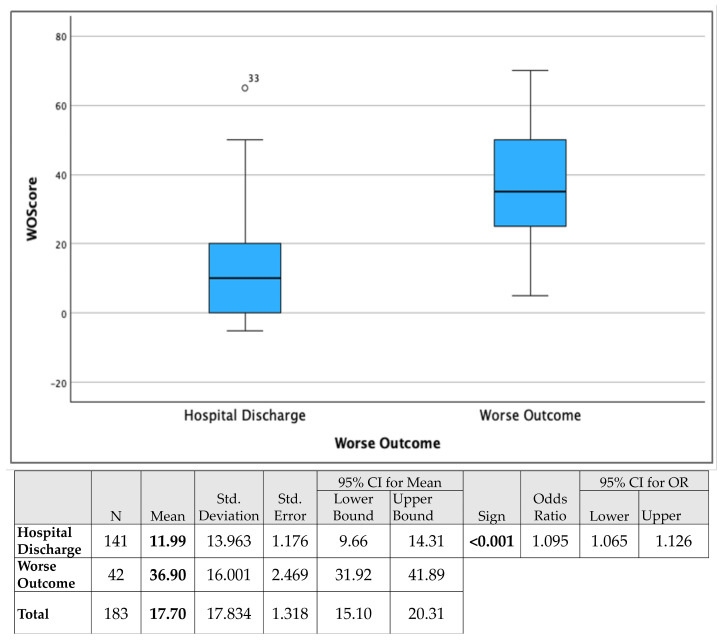
Boxplots illustrating WOScore distribution in the validation cohort, stratified by patient outcomes (hospital discharge vs. worse outcome). Patients with worse outcome demonstrated significantly higher WOScore values compared with those discharged without adverse events (*p* < 0.001). The accompanying table presents descriptive statistics, 95% confidence intervals, and logistic regression results, showing that each 1-point increase in WOScore was associated with a 9.5% increase in the odds of a worse outcome (OR = 1.095, 95% CI: 1.065–1.126, *p* < 0.001).

In the validation cohort, patients who experienced a worse outcome had a mean WOS of 36.90 ± 16.00, while those successfully discharged had a mean WOS of 11.99 ± 13.96. The Mann–Whitney U test confirmed a statistically significant difference in WOS distributions between the two outcome groups (U = 677.000, *p* < 0.001) (Figure 8). This finding replicated the associations observed in the derivation cohort, further confirming the discriminative capacity of the score. The logistic regression analysis shows that for each 1-point increase in WOScore, the odds of ICU readmission or in-hospital mortality increase by 9.5% (OR: 1.095, 95% CI: 1.065–1.126, *p* < 0.001).

#### 3.5.3. Discriminative Ability, Calibration and Cut-Off Analysis of WOS

**Figure 9:**
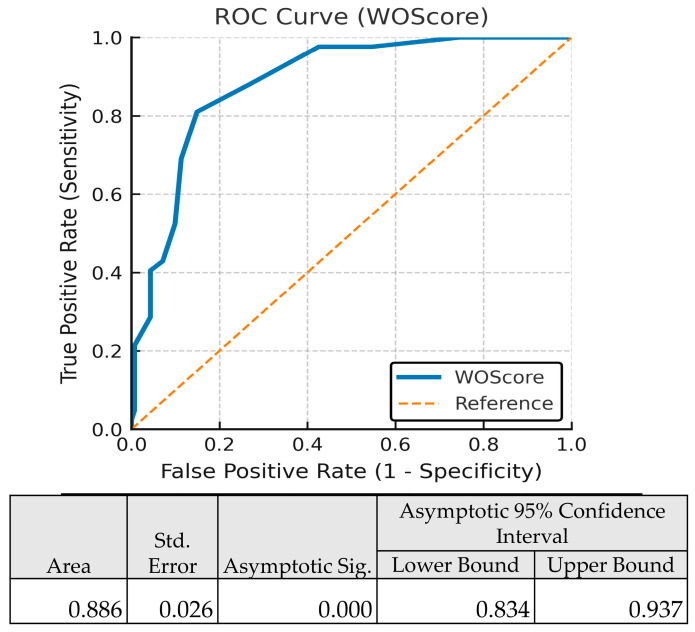
ROC Curve for WOScore Predicting Worse Outcome.

When applied as-is, without recalibration, the WOS maintained excellent discriminative performance in the validation cohort. The WOS demonstrated an AUC of 0.886 (95% CI: 0.834–0.937, *p* < 0.001), confirming strong ability to distinguish patients at risk of adverse post-ICU outcomes (Figure 9).

Calibration was also preserved, with a non-significant Hosmer–Lemeshow test (*p* = 0.771), a calibration slope of ~1.00003 and intercept near zero, and a Nagelkerke R^2^ of 0.52. Importantly, overall predictive accuracy was excellent, with a Brier score essentially equal to 1.042 and a Brier Skill Score of 0.41, indicating near-perfect calibration and strong overall performance in the validation cohort (Figure S4).

Using the prespecified cut-off score of 20 points, the WOS achieved a sensitivity of 88.1%, a specificity of 73.0%, a Positive Predictive Value (PPV) of 49.3% and a Negative Predictive Value (NPV) of 95.4% (Table 7). Patients with WOS ≥ 20 were at substantially higher risk, with an odds ratio of 20.06 (95% CI: 7.34–54.81, *p* < 0.001).

Decision Curve Analysis (DCA) further demonstrated that the application of the WOS yielded net clinical benefit across a wide range of probability thresholds, outperforming both “treat all” and “treat none” strategies (Supplementary Materials: Table S10).

### 3.6. Comparative Performance of Risk Scores

In the validation cohort, the WOScore consistently outperformed both SAPS III and SAPS II across all evaluated metrics (Table 8).

Discrimination was highest for the WOScore (AUC = 0.886), compared with SAPS III (0.797) and SAPS II (0.762), and this was accompanied by superior explained variance (Nagelkerke R^2^ = 0.521 vs. 0.450 and 0.349, respectively). Calibration was excellent for the WOScore (HL *p* = 0.732), while SAPS II demonstrated acceptable but weaker performance (HL *p* = 0.978), and SAPS III showed significant miscalibration (HL *p* = 0.001). Accuracy indices further supported the advantage of the WOScore, with the lowest Brier score (0.104) and highest Brier Skill Score (0.411).

## 4. Discussion

ICU readmission remains a major clinical and economic challenge, being associated with increased morbidity, extended hospital stays, and elevated healthcare costs. This is further confirmed by our study, where patients requiring ICU readmission demonstrated prolonged hospital stays and worse overall outcomes compared to those who were successfully discharged without requiring readmission, highlighting the clinical importance of early identification of high-risk individuals [4,8,26,27].

Several risk factors for ICU readmission have been identified in previous research, aligning with our findings, including comorbidities such as diabetes mellitus higher severity scores at ICU admission (SAPS III), and clinical conditions during ICU stay, including VAP and CRBSI. These findings underline that patient vulnerability, initial severity, and complications during ICU stay remain central determinants of poor outcomes. Conversely, some factors previously linked to increased ICU readmission risk were not confirmed in our cohort. These include discharge during holidays or out-of-hours, longer ICU length of stay, and the presence of a tracheostomy [28,29,30].

Importantly, we identified overlapping risk factors for both ICU readmission and in-hospital mortality, confirming that these adverse outcomes often reflect persistent physiological instability and residual organ dysfunction. This overlap and their clinical and economic burden justify analyzing them under the combined entity of “worse outcome,” which may provide a more integrated perspective for clinical decision-making and post-ICU planning [2,31].

Given these considerations, it is essential to analyze risk factors for worse outcome as a distinct entity, rather than evaluating ICU readmission and in-hospital mortality separately. This approach allows for a more comprehensive understanding of the determinants of post-ICU adverse events, facilitating the development of a simple yet reliable predictive model. Such a model could be integrated into structured ICU discharge and transition programs, enabling the early identification of high-risk patients and targeted post-ICU interventions. By focusing healthcare efforts on this vulnerable group, transition programs could be more cost-effective and efficient, minimizing unnecessary interventions while maximizing their impact on patient outcomes.

Our findings demonstrate that WOScore outperforms traditional severity scores such as SAPS II, SAPS III, and APACHE II, which have been used extensively for estimation of ICU prognosis at ICU admission but show limited performance in predicting post-ICU outcomes. Likewise, post-discharge tools such as SWIFT and RISC, although specifically developed for ICU readmission, have shown variable discriminative ability and are rarely used in routine practice due to complexity or poor external validation [14,25,32].

These models have shown comparable predictive performance with WOScore, with studies reporting AUC values ranging from 0.50 to 0.91 for post-ICU risk stratification [23]. However, unlike the majority of previous models, WOScore was designed as a simplified screening tool, combining routinely collected variables from admission, ICU stay and discharge providing a simple, bedside-applicable tool that demonstrates equal or superior predictive performance.

A key advantage of WOScore is its ability to reduce clinical workload and healthcare costs by efficiently stratifying post-ICU patients. WOScore not only showed statistically higher AUC values compared to other scores, but—more importantly—it demonstrated strong negative predictive value, excellent calibration, and clinical usability. These characteristics make it particularly valuable as a reliable screening tool, identifying low-risk patients who may require less intensive follow-up. This clinical significance is arguably more relevant for daily practice than numerical differences in AUC alone.

A key advantage of WOScore is its potential to guide structured ICU discharge and transition programs. By effectively ruling out low-risk individuals, it allows healthcare teams to allocate resources more efficiently and concentrate on patients with the greatest need for post-ICU support. This is particularly relevant in resource-constrained environments, where targeted use of limited personnel and facilities is essential. At the same time, we acknowledge that its application as a screening tool requires careful ethical consideration to avoid under-monitoring vulnerable individuals, as well as an assessment of the potential economic implications of widespread implementation. Therefore, while WOScore shows promise as a cost-effective and scalable tool, its integration into clinical pathways must be guided by further evidence.

This study has several limitations. Firstly, it is a single-center study conducted in a mixed ICU of a tertiary hospital, which may limit the generalizability of the findings to other ICU settings or healthcare systems. Secondly, as a partly retrospective study, data collection was limited to available electronic health records, potentially omitting important clinical information regarding patient status in general wards. Thirdly, the sample size of the validation cohort was relatively small, leading to a limited number of ICU readmissions and in-hospital deaths; however, the results remain statistically valid and comparable to previous studies in the field. Fourthly, the dichotomization of certain continuous variables (e.g., SAPS II > 26, WBC count > 13,000) may have resulted in a loss of predictive information, potentially underestimating the discriminatory ability of the model. Finally, follow-up was restricted to in-hospital outcomes, which limits the applicability of the WOScore to long-term prognosis after discharge. Further research is needed to evaluate the impact of WOScore within structured transition programs following ICU discharge, ideally with extended follow-up periods. Prospective, multi-center studies should investigate whether the implementation of WOScore-guided interventions can improve patient outcomes and reduce post-ICU complications. Additionally, integrating WOScore into digital health systems may enhance real-time risk stratification and facilitate personalized post-ICU care pathways. If validated in broader populations, WOScore could serve as a key component in post-ICU follow-up planning, optimizing both patient post-ICU outcomes and resource allocation.

## 5. Conclusions

This study highlights that ICU readmission and in-hospital mortality are driven by comorbidities, severity of illness, and ICU-acquired complications. The novel Worse Outcome Score (WOScore) integrates these elements into a simple, pragmatic and accurate predictive tool for post-ICU. Its strong calibration, high negative predictive value, and ease of use at discharge make it particularly valuable for supporting ICU transition programs and optimizing resource allocation. Future research should focus on multicenter validation, assessment of its clinical and economic impact, and integration into digital decision-support systems to facilitate its implementation in routine practice.

## Figures and Tables

**Table 1:** Independent predictors of ICU readmission (derivation cohort).

Variable	Univariate OR (95% CI)	*p*-Value	Multivariate OR (95% CI)	*p*-Value
Diabetes Mellitus	1.742 (1.182–2.57)	0.005	1.569 (1.182–2.507)	0.047
SAPS III on ICU admission	1.035 (1.021–1.049)	<0.001	1.022 (1.002–1.042)	0.029
VAP during ICU stay	2.702 (1.75–4.173)	<0.001	1.749 (1.040–2.943)	0.035
CRBSI during ICU stay	3.684 (2.360–5.751)	<0.001	2.520 (1.494–4.251)	<0.001
WBC count at ICU discharge	1.046 (1.022–1.093)	0.003	1.050 (1.017–1.085)	0.003

**Table 2:** Independent predictors of in-hospital mortality after ICU discharge (derivation cohort).

Variable	Univariate OR (95% CI)	*p*-Value	Multivariate OR (95% CI)	*p*-Value
Medical admission	2.224 (1.594–3.104)	<0.001	2.247 (1.294–3.904)	0.004
Lactate at ICU admission	1.009 (1.003–1.015)	0.002	1.014 (1.005–1.023)	0.003
Lactate clearance at 48 h	1.514 (1.132–2.026)	0.005	1.750 (1.070–2.864)	0.026
AKI during ICU stay	3.529 (2.568–4.848)	<0.001	1.671 (1.005–2.777)	0.048
Transfusion	3.685 (2.663–5.100)	<0.001	2.240 (1.360–3.690)	0.002
CRBSI during ICU stay	4.460 (3.015–6.598)	<0.001	1.947 (1.103–3.437)	0.021
Tracheostomy at discharge	8.412 (5.986–11.821)	<0.001	3.956 (2.275–6.879)	<0.001
GCS at discharge	0.764 (0.730–0.799)	0.001	0.882 (0.817–0.951)	0.001
SAPS III at admission	1.098 (1.081–1.115)	<0.001	1.046 (1.024–1.069)	<0.001

**Table 3:** Risk factors for worse outcome (ICU readmission or in-hospital death).

	Univariate Analysis			Multivariate Analysis		
	OR	95% CI	*p* Value	OR	95% CI	*p* Value
**Patients’ Characteristics**						
Age	1.027	1.019–1.036	<0.001	0.993	0.976–1.011	0.453
Heart Failure	1.451	1.008–2.088	0.045	0.883	0.527–1.480	0.638
CAD	1.424	1.029–1.972	0.033	0.809	0.504–1.297	0.378
Metastatic Cancer	2.697	1.585–4.592	<0.001	1.284	0.534–3.089	0.577
Diabetes Mellites	2.161	1.637–2.853	<0.001	1.627	1.084–2.443	0.019
Charlson Comorbidity Index	1.224	1.166–1.285	<0.001	1.107	0.977–1.254	0.111
**Category of Admission**						
Medical	1.977	1.512–2.586	<0.001	1.611	1.056–2.458	0.027
Elective Surgery	0.418	0.261–0.670	<0.001			
Emergent Surgery	0.686	0.501–0.939	0.019			
Trauma	0.608	0.399–0.926	0.020			
Neurological/Neurosurgical	1.534	1.146–2.055	0.004	1.187	0.715–1.971	0.507
**Patients’ origin**						
Ward/Other ICU	1.785	1.376–2.315	<0.001	1.012	0.665–1.540	0.956
Operating Room	0.536	0.395–0.728	<0.001			
Pre-ICU in-hospital days	1.030	1.019–1.041	<0.001	1.012	1.000–1.024	0.054
**Patients’ clinical condition at ICU admission**						
Respiratory Failure	1.330	1.000–1.768	0.050	0.959	0.594–1.547	0.863
Sepsis	1.755	1.249–2.464	0.001	0.894	0.515–1.553	0.691
SAPS II	1.061	1.050–1.071	<0.001			
SAPS III	1.092	1.078–1.107	<0.001	1.049	1.030–1.069	<0.001
SOFA	1.221	1.165–1.281	<0.001			
qSOFA	1.808	1.517–2.156	<0.001			
APACHE II	1.115	1.094–1.136	<0.001			
APACHE IV	1.040	1.033–1.047	<0.001			
GFR	0.991	0.988–0.995	<0.001	1.003	0.997–1.009	0.285
**Data from the Patient’s ICU Stay**						
ICU LOS	1.035	1.023–1.047	<0.001	0.985	0.962–1.008	0.207
Duration of MV	1.002	1.002–1.003	<0.001	1.000	0.998–1.001	0.767
Duration of vasopressors adm.	1.071	1.051–1.090	<0.001	1.001	0.986–1.017	0.853
Lactate Clearance 48 h	1.449	1.120–1.875	0.005	1.285	0.848–1.949	0.237
Transfusion	2.959	2.272–3.854	<0.001	1.697	1.122–2.567	0.012
Number of Blood products	1.085	1.050–1.122	<0.001	1.010	0.964–1.059	0.668
Infection in ICU	2.055	1.570–2.689	<0.001	1.249	0.790–1.975	0.342
VAP in ICU	4.638	3.288–6.542	<0.001	1.800	1.094–2.962	0.021
CRBSI in ICU	7.048	4.769–10.418	<0.001	4.349	2.516–7.516	<0.001
AKI	3.044	2.332–3.973	<0.001	1.066	0.677–1.678	0.783
CRRT	2.872	2.114–3.901	<0.001	0.852	0.466–1.558	0.603
Duration of CRRT	1.004	1.003–1.006	<0.001	1.001	0.998–1.003	0.580
Total Parenteral Nutrition	2.530	1.847–3.465	<0.001	1.404	0.892–2.209	0.143
**Patients’ Clinical Condition at ICU discharge**						
Tracheostomy	5.359	4.054–7.085	<0.001	2.433	1.510–3.919	<0.001
GCS	0.795	0.761–0.830	<0.001	0.968	0.885–1.059	0.473
SAPS II	1.101	1.085–1.117	<0.001	1.037	1.007–1.067	0.014
APACHE II	1.187	1.156–1.218	<0.001			
SOFA	1.311	1.230–1.389	<0.001			
GFR	0.999	0.996–1.002	0.520			
WBC	1.039	1.016–1.064	0.001	1.055	1.024–1.088	<0.001
HgB	0.722	0.657–0.795	<0.001	0.936	0.817–1.073	0.342
Lactate	1.094	1.066–1.124	<0.001	1.027	0.991–1.064	0.144

Variables that showed statistically significant differences between the outcome groups. Results from univariate and multivariate analyses are displayed, including odds ratios (OR), 95% confidence intervals (95% CI), and *p*-values.

**Table 4:** Worse Outcome Score (WOS) criteria and point allocation.

Worse Outcome Score		
**Medical episode**	No = −5 pts	Yes = 5 pts
**Diabetes Meletus** Patient’s comorbidity	No = 0 pts	Yes = 10 pts
**SAPS III** On patient’s ICU admission	≤70 = 0 pts	>70 = 5 pts
**Transfusion** Even 1 unit blood products during ICU stay	No = 0 pts	Yes = 10 pts
**VAP** OR **CRBSI** During patient’s ICU stay	No = 0 pts	Yes = 20 pts
**SAPS II** On ICU discharge	≤26 = 0 pts	>26 = 5 pts
**Tracheostomy** On patient’s ICU discharge	No = 0 pts	Yes = 15 pts
**WBC** On patient’s ICU discharge	≤13 K/μL = 0 pts	>13 K/μL = 5 pts

**Table 5:** Comparison of Worse Outcome Score (WOS) between patients who were successfully discharged and those who experienced ICU readmission or in-hospital death.

	N	Mean	Std. Deviation	Std. Error	95% Confidence Interval for Mean		Sign	Odds Ratio	95% CI for OR	
					Lower Bound	Upper Bound			Lower	Upper
Hospital Discharge	872	15.67	14.652	0.0496	14.70	16.65	<0.001	1.082	1.072	1.093
Worse Outcome	318	38.55	17.919	1.005	36.58	42.20				
Total	1190	21.79	18.589	0.0539	20.73	22.85				

**Table 6:** Classification performance of the Worse Outcome Score (WOS) using a cut-off of 20.

			Worse Outcome		
			Hospital Discharge	Readmission or In-Hospital Death	Total
WOScore	<20	n	533	42	575
		(%)	61.1%	13.2%	48.32%
	≥20	n	339	276	615
		(%)	38.9%	86.8%	51.68%
Total		n	872	318	1190
		(%)	100%	100%	100%
		Sensitivity		Specificity	
		86.8%		61.1%	
		PPV (Positive Predictive Value)		NPV (Negative Predictive Value)	
		44.9%		92.7%	

**Table 7:** Classification Performance of WOScore for Predicting Worse Outcome.

			Worse Outcome		
			Hospital Discharge	Readmission or In-Hospital Death	Total
WOScore	<20	n	103	5	108
		(%)	73.0%	11.9%	59.02%
	≥20	n	38	37	75
		(%)	27.0%	88.1%	40.98%
Total		n	141	42	183
		(%)	100%	100%	100%
		Sensitivity		Specificity	
		88.1%		73.0%	
		PPV (Positive Predictive Value)		NPV (Negative Predictive Value)	
		49.3%		95.4%	

**Table 8:** Comparative performance of the WOScore, SAPS III, and SAPS II in the validation cohort.

Score	AUC	Brier Score	Nagelkerke R^2^	Calibration Slope	Hosmer–Lemeshow Test *p*-Value
WOScore	0.886	0.1042	0.521	1.0003	0.7323
SAPS III (ICU admission)	0.797	0.1064	0.45	1.0004	0.0011
SAPS II (ICU discharge)	0.762	0.1273	0.349	1.0007	0.9781

## Data Availability

The original contributions presented in this study are included in the article/supplementary material. Further inquiries can be directed to the corresponding author(s).

## Supplementary Materials

The following supporting information can be downloaded at: https://www.mdpi.com/article/10.3390/jpm15100479/s1, Figure S1: Distribution of reasons for ICU readmission; Figure S2: Receiver operating characteristic (ROC) curves for predicting ICU readmission using clinical severity scores at ICU admission (Figure S2A) and ICU discharge (Figure S2B); Figure S3: Receiver operating characteristic (ROC) curves for predicting in-hospital mortality using clinical severity scores at ICU admission (Figure S3A) and ICU discharge (Figure S3B); Figure S4: Calibration curve (validation cohort); Table S1: Determination of Hospital-Acquired Infections During ICU Stay; Table S2: Collected variables categorized for analysis. Selection was based on clinical relevance and existing literature; Table S3: Descriptive characteristics of ICU patients, stratified by readmission status; Table S4: Univariate and multivariate analysis of risk factors for ICU readmission (odds ratios (OR), 95% confidence intervals (95% CI), and *p*-values of analyzed factors; Table S5: Descriptive characteristics of ICU patients stratified by in-hospital mortality after ICU discharge; Table S6: Univariate and multivariate analysis of risk factors for in-hospital mortality of patients discharged from ICU; Table S7: Descriptive characteristics of ICU patients, stratified by worse outcome; Table S8: Net benefit of the predictive model compared with treat-all and treat-none strategies across selected threshold probabilities; Table S9: Comparison of Baseline Characteristics Between Derivation and Validation Cohorts; Table S10: Net benefit of the predictive model across selected threshold probabilities in the validation cohort.
