# Supplementary material for: ICU Readmission and In-Hospital Mortality Rates for Patients Discharged from the ICU—Risk Factors and Validation of a New Predictive Model: The Worse Outcome Score (WOScore)
Source: J Pers Med. 2025 Oct 3;15(10):479. doi: 10.3390/jpm15100479 (PMC12565189; doi:10.3390/jpm15100479)
Supplement: Supplementary file 1 [file jpm-15-00479-s001.zip › jpm-3800562-supplementary.pdf]

Supplementary Material (Supplement)

Table S1: Determination of Hospital-Acquired Infections During ICU Stay

**Hospital-Acquired Infection (HAI)**	HAI is considered present if, after the initial 48 hours of ICU admission, the patient exhibits at least one of the following: (a) a new episode of fever accompanied by hemodynamic instability; (b) positive cultures obtained post the second 24 hours of ICU stay, with initial admission cultures being negative; or (c) escalation of antibiotic therapy based on clinical or laboratory indicators suggestive of infection
**Catheter-related bloodstream infection (CRBSI)**	CRBSI is diagnosed when there is evidence of identical microbial strains from both peripheral blood cultures and catheter tip cultures, or when differential time to positivity (DTP) indicates the catheter as the infection source. These criteria are outlined in the CDC's guidelines for the prevention of intravascular catheter-related infections
**Ventilator - associated pneumonia (VAP)**	VAP is defined as pneumonia occurring in patients who have been on mechanical ventilation for more than 48 hours. Diagnosis requires a combination of radiographic, clinical, and laboratory findings: (a) new or progressive infiltrates on chest imaging; (b) at least one systemic sign such as fever (>38°C), leukocytosis (>12,000 cells/mm^3), or leukopenia (<4,000 cells/mm^3); and (c) at least two pulmonary criteria, including purulent respiratory secretions, increased respiratory secretions, or worsening gas exchange

Table S2: Collected variables categorized for analysis. Selection was based on clinical relevance and existing literature

Domain	Variables
**Demographics**	Age, Gender, BMI
**Comorbidities**	Charlson Comorbidity Index, Heart Failure, CAD, COPD, CKD, Metastatic Cancer, Diabetes Mellitus
**ICU Admission Characteristics**	Reason for ICU admission, Source of admission, Days in hospital before ICU, Admission Category
**ICU Clinical Course**	ICU LOS, Duration of Mechanical Ventilation, Vasopressors, Blood products transfusions, CRRT, Delirium, VAP, CRBSI, AKI, TPN
**Discharge Status**	Tracheostomy, GCS at discharge, Off-hour/weekend discharge, GFR at discharge
**Laboratory Admission Values**	Lactate, Creatinine, GFR
**Laboratory Discharge Values**	WBC, Hemoglobin, Platelets, Lactate, CRP, PaO2/FiO2

**Severity Scores (Admission)**	SAPS III, APACHE II, APACHE IV, SOFA, qSOFA
**Severity Scores (Discharge)**	SAPS II SOFA, qSOFA

Table S3. Descriptive characteristics of ICU patients, stratified **by readmission status**.
P-values were calculated using the **independent t-test** for normally distributed continuous variables, the **chi-square test** for categorical variables, and the **Mann-Whitney U test** for non-normally distributed continuous variables. Data are presented as median (IQR), mean (SD), or frequency (%), depending on their distribution.

**Characteristics**	**Total patients n=1190 patients**	**Non readmitted patients n= 1064**	**Readmitted patients n= 126**	**P-value**
**Patients' characteristics**				
Age (IQR)	64 (50-74)	65 (51 - 75)	66 (51 - 75)	0.338
Male gender (%)	757 (63.6)	676 (63.5)	81 (64.3)	0.868
BMI (IQR)	28.1 (24.3 – 32.3)	28.1 (24.2 – 32.3)	28.4 (24.7 – 32.9)	0.427
**Comorbidities**				
Heart Failure (%)	152 (12.8)	137 (12.9)	15 (11.9)	0.752
CAD (%)	204 (17.1)	177 (16.6)	27 (21.4)	0.177
COPD (%)	198 (16.7)	181 (17.0)	17 (13.6)	0.333
CKD (%)	110 (9.3)	95 (8.9)	15 (11.9)	0.277
Metastatic Cancer (%)	58 (4.9)	47 (4.4)	11 (8.7)	0.034
Diabetes Mellites (%)	310 (26.1)	264 (24.8)	46 (36.5)	0.005
Charlson Comorbidity Index (IQR)	3 (1.0 – 5.0)	4 (1.0 – 5.0)	4 (2.0 – 6.0)	0.014
**Category of Admission**				
**Medical** (%)	652 (54.8)	575 (54.0)	77 (61.1)	0.132
Elective Surgery (%)	153 (12.9)	141 (13.3)	12 (9.5)	0.232
Emergent Surgery (%)	293 (24.6)	266 (25.0)	27 (21.4)	0.379
**Trauma** (%)	157 (13.2)	142 (13.3)	15 (11.9)	0.651
Neurological/ Neurosurgical (%)	274 (23.0)	245 (23.0)	29 (23.0)	0.998
**Patients' origin**				
Ward / Other ICU (%)	459 (38.6)	395 (37.1)	64 (50.8)	0.003
ED / Other hospital (%)	379 (31.8)	343(32.2)	36 (28.6)	0.404
Operating Room (%)	352 (29.6)	326 (30.6)	26 (20.6)	0.020
Pre -ICU in-hospital days (SD)	6.16 (16.21)	5.72 (16.17)	9.92 (16.07)	0.006
**Patients' clinical condition at ICU admission**				
Respiratory Failure (%)	309 (26.0)	273 (25.7)	36 (28.6)	0.481
Sepsis (%)	173 (14.5)	144 (13.5)	29 (23.0)	0.004
SAPS II (IQR)	38 (27 – 49)	38 (28 - 50)	41 (32 - 50)	0.019
SAPS III (SD)	71.19 (13.52)	70.52 (13.44)	76.82 (12.89)	< 0.001
SOFA (IQR)	8 (6 – 10)	8 (7 -10)	9 (6 - 11)	0.178
qSOFA (SD)	1.76 (0.81)	1.76 (0.80)	1.77 (0.85)	0.912
APACHE II (IQR)	17 (12 – 23)	18 (12 - 23)	19 (14 -24)	0.007
APACHE IV (IQR)	56 (41 – 72)	57 (42 - 74)	60 (47 – 77.5)	0.005
Lactate (mg/dl) (IQR)	18.6 (12.0 – 33.0)	19.0 (12.0 – 33.1)	18.4 (11.0 – 30.9)	0.495

GFR (ml/min/1,73m[2]) (IQR)	66.1 (41.6 – 90.5)	66.0 (41.0 – 90.4)	59 (39.1 – 85.9)	0.159
**Data from the Patient's ICU Stay**				
ICU LOS (IQR)	7 (4 – 15)	8 (4 - 16)	9 (5 -18)	0.003
Duration of MV (h) (IQR)	74 (18.0 - 237)	96 (25 - 265)	138 (26 - 285)	0.036
Duration of vasopressors administration (24h) (IQR)	4 (2 - 8)	4 (2 – 9)	4 (2 -10)	0.126
Lactate Clearance 24h (IQR)	-0.34 (-0.56 – 0.0)	-0.34 (-0.56 – 0.0)	-0.25 (-0.54 – 0.2)	0.282
Lactate Clearance 48h (IQR)	-0.40 (-0.66 – 0.1)	-0.39(-0.65 – (-0.8))	-0.38 (-0.63 – 0.0)	0.349
Transfusion (%)	469 (39.4)	409 (38.4)	60 (47.6)	0.046
Number of Blood products (IQR)	0 (0 -2)	0 (0 - 2)	0 (0 -3)	0.077
Infection in ICU (%)	360 (30.3)	309 (29.0)	51 (40.5)	0.008
VAP in ICU (%)	162 (13.6)	128 (12.0)	34 (27.0)	< 0.001
CRBSI in ICU (%)	132 (11.1)	98 (9.2)	34 (27.0)	< 0.001
AKI (%)	398 (33.4)	344 (32.3)	54 (42.9)	0.018
CRRT (%)	217 (18.2)	182 (17.1)	35 (27.8)	0.003
Duration of CRRT (h) (SD)	28.31 (96.81)	24.86 (89.22)	57.20 (143.00)	0.014
Delirium (%)	356 (29.9)	322 (30.3)	34 (27.0)	0.447
Total Parenteral Nutrition (%)	202 (17.0)	169 (15.9)	33 (26.2)	0.004
**Patients' Clinical Condition at ICU discharge**				
Holidays / Out of hours (%)	214 (18.0)	193 (18.1)	21 (16.7)	0.684
Tracheostomy (%)	336 (28.2)	293 (27.5)	43 (34.1)	0.120
Mechanical Ventilation (%)	50 (4.2)	48(4.5)	2 (1.6)	0.121
GCS (SD)	13.66 (2.94)	13.67 (2.96)	13.62 (2.75)	0.864
SAPS II (IQR)	24 (16 - 31)	24 (16 - 32)	28 (21 - 33)	< 0.001
APACHE II (IQR)	11 (7 - 16)	11 (7 - 16)	13 (10 - 17)	< 0.001
SOFA (IQR)	3 (2 - 5)	3 (2 - 5)	4 (3 - 5)	0.003
qSOFA (SD)	1.07 (0.78)	1.08 (0.79)	1.04 (0.69)	0.620
GFR (ml/min/1.73m[2]) (IQR)	83.7 (52.4 – 115.5)	84.6 (52.8 – 116.8)	81.3 (49.4 – 117.5)	0.977
WBC (K/μl) (IQR)	11.0 (8.3 – 14.3)	10.9 (8.3 – 13.9)	12.3 (9.0 – 16.3)	0.012
HgB (g/dl) (IQR)	9.3 (8.5 – 14.3)	9.3 (8.5 – 10.6)	9.1 (8.4 – 10.0)	0.005
Lactate (mg/dl) (IQR)	9.6 (7.0 – 13.0)	9.3 (7.0 – 13.0)	10 (8 - 14)	0.008
CRP (mg/dl) (IQR)	7.44 (3.23 – 14.03)	7.5 (3.3 – 13.9)	7.1 (2.8 – 15.3)	0.621
PaO_2/FiO_2 ratio (IQR)	247 (184.5 – 317.3)	249.5 (187 - 315)	250 (171 - 334)	0.616

Table S4. Univariate and multivariate analysis of risk factors for **ICU readmission** (odds ratios (OR), 95% confidence intervals (95% CI), and p-values of analyzed factors).

	Univariate Analysis			Multivariate Analysis		
	**OR**	**95% CI**	**P value**	**OR**	**95% CI**	**P value**
**Patients' characteristics**						
Metastatic Cancer	2.070	1.044 – 4.103	0.034	1.903	0.800 – 4,622	0.095
Diabetes Mellites	1.742	1.182 – 2.570	0.005	1.569	1.182 – 2.507	0.047
Charlson Comorbidity Index	1.092	1.023 – 1.165	0.008	0.985	0.882 – 1.101	0.794
**Patients' origin**						
Ward / Other ICU	1.741	1.202 – 2.523	0.003	1.406	0.906 – 2.181	0.128
Operating Room	0.591	0.377 – 0.928	0.022			
Pre -ICU in-hospital days	1.010	1.001 – 1.019	0.027	1.004	0.995 – 1.013	0.359
**Patients' clinical condition at ICU admission**						
Sepsis	1.910	1.217 – 2.997	0.005	1.381	0,764 – 2.495	0.285
SAPS II	1.014	1.002 – 1.026	0.024			
SAPS III	1.035	1.021 – 1.049	<0.001	1.022	1.002 – 1.042	0.029
APACHE II	1.026	1.004 – 1.048	0.022			
APACHE IV	1.010	1.002 – 1.018	0.012			
**Data from the Patient's ICU Stay**						
ICU LOS	1.008	0.999 – 1.018	0.078			
Duration of MV	1.001	1.000 – 1.001	0.072			
Transfusion	1.456	1.005 – 2.109	0.047	0.976	0.867 – 1.279	0.357
Infection in ICU	1.654	1.132 – 2.417	0.009	0.890	0.542 – 1.462	0.646
VAP in ICU	2.702	1.750 – 4.173	<0.001	1.749	1.040 – 2.943	0.035
CRBSI in ICU	3.684	2.360 – 5.751	<0.001	2.520	1.494 – 4.251	<0.001
AKI	1,570	1.078 – 2.285	0.019	0.897	0.421 – 1.255	0.252
CRRT	1.864	1.223 – 2.840	0.004	1.072	0.529 – 2.172	0.846
Duration of CRRT	1.002	1.001 – 1.004	0.001	1.001	0.999 – 1.003	0.332
Total Parenteral Nutrition	1.879	1.223 – 2.888	0.004	1.180	0.713 – 1.954	0.520
**Patients' Clinical Condition at ICU discharge**						
SAPS II	1.025	1.010 – 1.039	<0.001			
APACHE II	1.055	1.025 – 1.085	<0.001	0.994	0.952 – 1.002	0.078
SOFA	1.084	1.007 – 1.168	0.032			
WBC (K/μl)	1.046	1.015 – 1.078	0.003	1.050	1.017 – 1.085	0.003
HgB (g/dl)	0.810	0.710 – 0.923	0.002	0.881	0.751 – 1.034	0.122
Lactate (mg/dl)	1.057	1.022 – 1.093	0.001	1.020	0.981 – 1.060	0.321

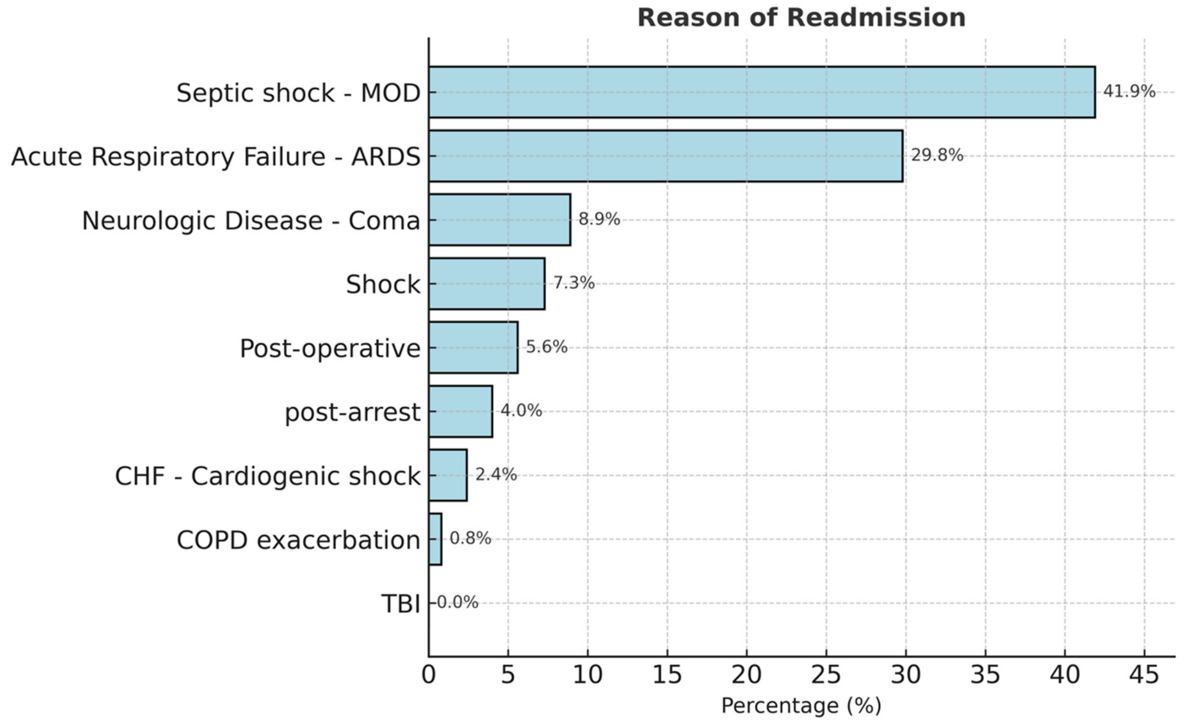

Figure S1. Distribution of reasons for ICU readmission.

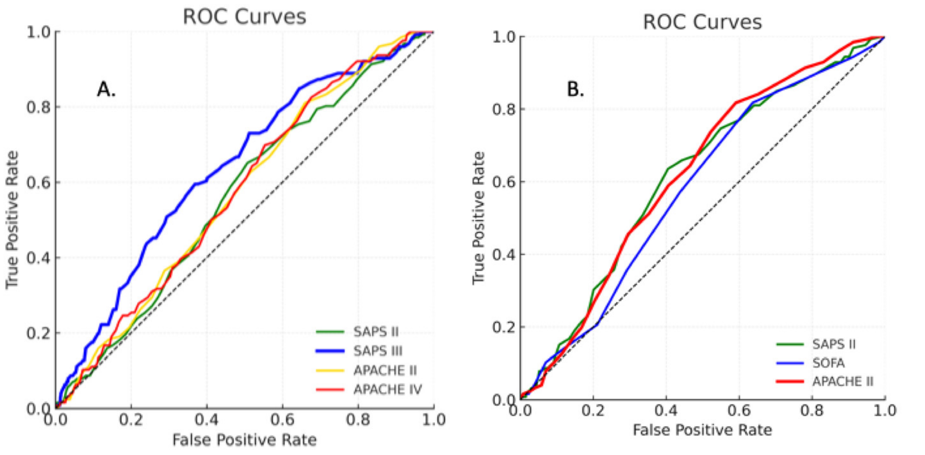

Area Under the ROC Curve – Readmission: Scores on ICU Admission			
	Area	Asymptomatic 95% Confidence Interval	
		Lower Bound	Upper Bound
**SAPS II**	0.564	0.514	0.613
**SAPS III**	0.639	0.589	0.688
**APACHE II**	0.573	0.525	0.622
**APACHE IV**	0.576	0.527	0.624

Area Under the ROC Curve – Readmission: Scores on ICU discharge			
	Area	Asymptomatic 95% Confidence Interval	
		Lower Bound	Upper Bound
**SAPS II**	0.610	0.561	0.658
**SOFA**	0.581	0.532	0.630
**APACHE II**	0.611	0.565	0.657

Figure S2. Receiver operating characteristic (ROC) curves for predicting **ICU readmission** using clinical severity scores at ICU admission (Figure S2A) and ICU discharge (Figure S2B)**.**

Table S5. Descriptive characteristics of ICU patients stratified by in-hospital mortality after ICU discharge. P-values were calculated using the **independent t-test** for normally distributed continuous variables, the **chi-square test** for categorical variables, and the **Mann-Whitney U test** for non-normally distributed continuous variables. Data are presented as median (IQR), mean (SD), or frequency (%), depending on their distribution

**Characteristics**	**Total discharged patients n=1190 patients**	**Patients admitted to ICU and discharged from hospital alive n= 998 patients**	**Patients died in hospital after ICU discharge n= 192 patients**	**P-value**
**Patients' characteristics**				
Age (IQR)	64 (50 - 74)	63 (48 - 73)	72 (62 - 79)	< 0.001
Male gender (%)	757 (63.6)	642 (64.3)	115 (59.9)	0.242
BMI (IQR)	28.1 (24.3 – 32.3)	28.3 (24.4 – 32.4)	28.0 (23.9 – 32.1)	0.751
Heart Failure (%)	152 (12.8)	116 (11.6)	36 (18.8)	0.007
CAD (%)	204 (17.1)	164 (16.4)	40 (20.8)	0.138
COPD (%)	198 (16.7)	159 (15.9)	39 (20.3)	0.137
CKD (%)	110 (9.3)	87 (8.7)	23 (12.0)	0.154
Metastatic Cancer (%)	58 (4.9)	41 (4.1)	17 (8.9)	0.005
Diabetes Mellites (%)	310 (26.1)	236 (23.6)	74 (38.5)	< 0.001
Charlson Comorbidity Index (IQR)	3 (1 - 5)	3 (1 - 5)	5 (3 - 6)	< 0.001
**Category of Admission**				
**Medical** (%)	652 (54.8)	516 (51.7)	136 (70.8)	< 0.001
**Surgical**				
Elective Surgery (%)	153 (12.9)	144 (14.4)	9 (4.7)	< 0.001
Emergent Surgery (%)	293 (24.6)	257(25.8)	36 (18.8)	0.039
**Trauma** (%)	157 (13.2)	142(14.2)	15 (7.8)	0.016
Neurological/ Neurosurgical (%)	274 (23.0)	212 (21.2)	62 (32.3)	< 0.001
**Patients' origin**				
Ward / Other ICU (%)	459 (38.6)	367 (36.8)	92 (47.9)	0.004
ED / Other hospital (%)	379 (31.8)	318 (31.9)	61 (31.8)	0.980
Operating Room (%)	352 (29.6)	313 (31.4)	39 (20.3)	0.002
Pre -ICU in-hospital days (SD)	6.16 (16.21)	5.17 (11.02)	11.32 (31.15)	0.007
**Patients' clinical condition at ICU admission**				
Respiratory Failure (%)	309 (26.0)	249 (24.9)	60 (31.3)	0.068
Sepsis (%)	173 (14.5)	138 (13.8)	35 (18.2)	0.113
SAPS II (IQR)	38 (27 - 49)	36 (27 - 46)	53 (41 - 61)	< 0.001
SAPS III (SD)	71.19 (13.52)	68.85 (12.68)	83.34 (10.99)	< 0.001
SOFA (IQR)	8 (6 - 10)	8 (6 - 10)	10 (8 - 12)	< 0.001
qSOFA (SD)	1.76 (0.81)	1.70 (0.80)	2.08 (0.75)	< 0.001
APACHE II (IQR)	17 (12 - 23)	16 (12 - 21)	24 (20 - 28)	< 0.001
APACHE IV (IQR)	56 (41 - 72)	53 (40 - 68)	78 (61 - 92)	< 0.001
Lactate (mg/dl) (IQR)	18.6 (12.0 – 33.0)	18.0 (11.7 – 32.0)	20.0 (30,29)	0.018
GFR (ml/min/1,73m^2) (IQR)	66.1 (41.6 – 90.5)	68.7 (43.2 – 92.9)	58,31 (13 – 36)	< 0.001
**Data from the Patient's ICU Stay**				

ICU LOS (IQR)	7 (4 - 15)	7 (4 - 14)	14 (7 - 23)	< 0.001
Duration of MV (h) (IQR)	74 (18 - 237)	82 (21 - 221)	233 (89 - 399)	< 0.001
Duration of vasopressors administration (24h) (IQR)	4 (2 - 8)	4 (2 - 8)	8 (4 - 15)	< 0.001
Lactate Clearance 24h (IQR)	-0,34 (-0.56 – 0.0)	-0,34 (-0.56 – 0.0)	-0.29 (-0.51 – 0.5)	0.304
Lactate Clearance 48h (IQR)	-0,40 (-0.66 – 0.1)	-0,40 (-0.66 – 0.0)	-0.36 (-0.63 – 0.9)	0.038
Transfusion (%)	469 (39.4)	343 (34.4)	126 (65.6)	< 0.001
Number of Blood products (IQR)	0 (0 - 2)	0 (0 – 1)	1 (0 – 3)	< 0.001
Infection in ICU (%)	360 (30.3)	277 (27.8)	83 (43.2)	< 0.001
VAP in ICU (%)	162 (13.6)	104 (10.4)	58 (30.2)	< 0.001
CRBSI in ICU (%)	132 (11.1)	78 (7.8)	54 (28.1)	< 0.001
AKI (%)	398 (33.4)	285 (28.6)	113 (58.9)	< 0.001
CRRT (%)	217 (18.2)	153 (15.3)	64 (33.3)	< 0.001
Duration of CRRT (h) (SD)	28.31 (96.81)	21.93 (81.22)	61.34 (150.19)	< 0,001
Delirium (%)	356 (29.9)	307 (30.8)	49 (25.5)	0.146
Total Parenteral Nutrition (%)	202 (17.0)	147 (14.7)	55 (28.6)	< 0.001
**Patients' Clinical Condition at ICU discharge**				
Holidays / Out of hours (%)	214 (18.0)	186 (18.6)	28 (14.6)	0.180
Tracheostomy (%)	336 (28.2)	204 (20.4)	132 (68.8)	< 0.001
Mechanical Ventilation (%)	50 (4.2)	33(3.3)	17 (8.9)	< 0.001
GCS (SD)	13.67 (2.94)	14.19 (2.22)	10.97 (4.41)	< 0.001
SAPS II (IQR)	24 (16 - 31)	22 (15 - 29)	34 (27 - 44)	< 0.001
APACHE II (IQR)	11 (7 - 16)	10 (7 - 15)	17 (13 - 21)	< 0.001
SOFA (IQR)	3 (2 - 5)	3 (2 - 4)	5 (4 - 7)	< 0.001
qSOFA (SD)	1.07 (0.78)	0.98 (0.75)	1.52 (0.77)	< 0.001
GFR (ml/min/1.73m^2) (IQR)	83.7 (52.4 – 115.5)	86.7 (55.3 – 117)	64.9 (45 - 109)	0.064
WBC (K/μl) (IQR)	11.0 (8.3 – 14.3)	10.8 (8.3 – 13.9)	12.5 (9.1 – 14.9)	0.017
HgB (g/dl) (IQR)	9,3 (8.5 – 14.3)	9.5 (8.5 – 10.7)	8.8 (8.3 – 9.6)	< 0.001
Lactate (mg/dl) (IQR)	9.6 (7.0 – 13.0)	9.0 (7.0 – 12.1)	12.0 (8.1 – 15.0)	< 0.001
CRP (mg/dl) (IQR)	7.44 (3.23 – 14.03)	7.63 (3.2 – 14.4)	6.8 (3.4 – 12.8)	0.232
PaO_2/FiO_2 ratio (IQR)	247 (184.5 – 317.3)	247 (182 - 314)	259 (204 - 331)	0.035

.

Table S6. Univariate and multivariate analysis of risk factors for in – hospital mortality of patients discharged from ICU. The table presents odds ratios (OR), 95% confidence intervals (95% CI), and p-values of various analyzed factors.

	Univariate Analysis			Multivariate Analysis		
	OR	95% CI	P value	OR	95% CI	P value
**Patients' characteristics**						
Age	1.038	1.027 – 1.050	<0.001	1.018	0.998 – 1.038	0.076
Heart Failure	1.741	1.155 – 2.626	0.008	1.067	0.586 – 1.945	0.832
Metastatic Cancer	2.252	1.251 – 4.054	0.007	1.790	0.667 – 4.803	0.248
Diabetes Mellites	2.005	1.449 – 2.775	<0.001	1.113	0.688 – 1.801	0.663
Charlson Comorbidity Index	1.243	1.175 – 1.315	<0.001	1.110	0.965 – 1.277	0.145
**Category of Admission**						
Medical	2.224	1.594 – 3.104	<0.001	2.247	1.294 – 3.904	0.004
Elective Surgery	0.326	0.169 – 0.632	<0.001			
Emergent Surgery	0.660	0.447 – 0.974	0.036			
Trauma	0.507	0.291 – 0.885	0.017			
Neurological/ Neurosurgical	1.805	1.289 – 2.529	<0.001	1.420	0.773 – 2.609	0.259
**Patients' origin**						
Ward / Other ICU	1.557	1.147 – 2.124	0.005	0.703	0.423 – 1.166	0.172
Operating Room	0.577	0.397 – 0.837	0.004			
Pre -ICU in-hospital days	1.020	1.010 – 1.031	<0.001	1.009	0.999 – 1.019	0.089
**Patients' clinical condition at ICU admission**						
SAPS II	1.077	1.064 – 1.091	<0.001			
SAPS III	1.098	1.081 – 1.115	<0.001	1.046	1.024 – 1.069	<0.001
SOFA	1.298	1.224 – 1.377	<0.001			
qSOFA	1.852	1.503 – 2.282	<0.001			
APACHE II	1.140	1.114 – 1.166	<0.001			
APACHE IV	1,049	1.040 – 1.057	<0.001			
Lactate	1.009	1.003 – 1.015	0.002	1.014	1.005 – 1.023	0.003
GFR	0.990	0.085 – 0.994	<0.001	1.007	0.998 – 1.016	0.610
**Data from the Patient's ICU Stay**						
ICU LOS	1.029	1.018 – 1.041	<0.001	0.973	0.922 – 1.027	0.327
Duration of MV	1.002	1.002 – 1.003	<0.001	1.001	0.998 – 1.003	0.608
Duration of vasopressors adm.	1.073	1.052 – 1.093	<0.001	1.012	0.989 – 1.036	0.315
Lactate Clearance 48h	1.514	1.132 – 2.026	0.005	1.750	1.070 – 2.864	0.026
Transfusion	3.685	2.663 – 5.100	<0.001	2.240	1.360 – 3.690	0.002
Number of Blood products	1.068	1.035 – 1.101	<0.001	1.022	0.967 – 1.080	0.438
Infection in ICU	1.952	1.422 – 2.679	<0.001	1.453	0.881 – 2.396	0.144
VAP in ICU	3.689	2.552 – 5.334	<0.001	1.348	0.778 – 2.335	0.287
CRBSI in ICU	4.460	3.015 – 6.598	<0.001	1.947	1.103 – 3.437	0.021
AKI	3.529	2.568 – 4.848	<0.001	1.671	1.005 – 2.777	0.048
CRRT	2.737	1.937 – 3.867	<0.001	0.749	0.374 – 1.502	0.416
Duration of CRRT	1.003	1.002 – 1.004	<0.001	0.998	0.995 – 1.001	0.175
Total Parenteral Nutrition	2.305	1.611 – 3.295	<0.001	1.446	0.853 – 2.449	0.170
**Patients' Clinical Condition at ICU discharge**						
Tracheostomy	8.412	5.986 – 11.821	<0.001	3.956	2.275 – 6.879	<0.001
Mechanical Ventilation	2.822	1.538 – 5.176	<0.001	1.125	0.488 – 2.594	0.781
GCS	0.764	0.730 – 0.799	0.001	0.882	0.817 – 0.951	0.001
SAPS II	1.101	1.084 – 1.118	<0.001	1.009	0.994 – 1.024	0.254
APACHE II	1.216	1.179 – 1.255	<0.001			
SOFA	1.348	1.263 – 1.438	<0.001			
WBC	1.021	0.994 – 1.049	0.132			
HgB	0.721	0.642 – 0.810	<0.001	1.011	0.852 – 1.200	0.897

Lactate	1.084	1.053 – 1.116	<0.001	1.026	0.985 – 1.069	0.212
PaO_2/FiO_2 ratio	1.002	1.000 – 1.003	0.081			

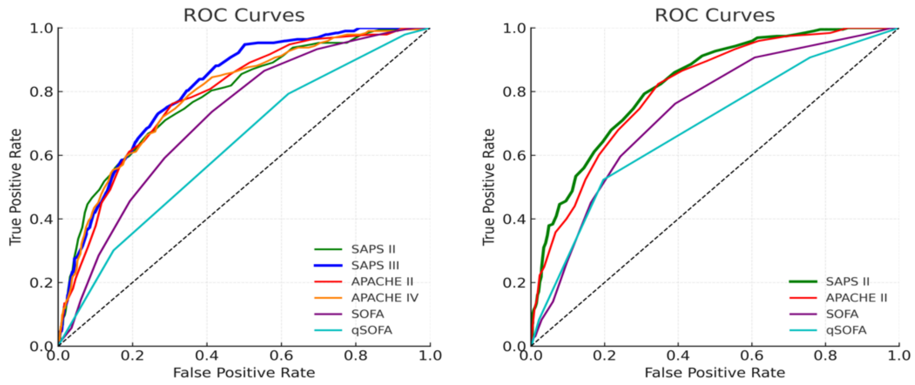

Area Under the ROC Curve: In – hospital mortality: Scores on ICU Admission			
	Area	Asymptomatic 95% Confidence Interval	
		Lower Bound	Upper Bound
**SAPS II**	0.786	0.751	0.822
**SAPS III**	0.811	0.782	0.841
**SOFA**	0.717	0.681	0.754
**qSOFA**	0.626	0.583	0.668
**APACHE II**	0.787	0.754	0.820
**APACHE IV**	0.781	0.755	0.823

Area Under the ROC Curve – In -hospital mortality: Scores on ICU discharge			
	Area	Asymptomatic 95% Confidence Interval	
		Lower Bound	Upper Bound
**SAPS II**	0.823	0.793	0.853
**SOFA**	0.736	0.699	0.772
**qSOFA**	0.689	0.647	0.731
**APACHE II**	0.809	0.778	0.837

Figure S3. Receiver operating characteristic (ROC) curves for predicting **in-hospital mortality** using clinical severity scores at ICU admission (Figure S3A) and ICU discharge (Figure S3B)**.**

Table S7. Descriptive characteristics of ICU patients, stratified by **worse outcome**. P-values were calculated using the **independent t-test** for normally distributed continuous variables, the **chi-square test** for categorical variables, and the **Mann-Whitney U test** for non-normally distributed continuous variables. Data are presented as median (IQR), mean (SD), or frequency (%), depending on their distribution.

**Characteristics**	**Total discharged patients n=1190 patients**	**Patients admitted to ICU and discharged from hospital without readmission n= 872 patients**	**Patients with worse outcome (readmission OR in-hospital death) after ICU discharge n= 318 patients**	**P-value**
**Patients' characteristics**				
Age (IQR)	64 (50 - 74)	62 (47 - 73)	70 (59 - 77)	< 0.001

Male gender (%)	757 (63.6)	561 (64.3)	196 (61.6)	0.392
BMI (IQR)	28.1(24.3 – 32.3)	28,88 (7.19)	28.4 (24.5 – 32.5)	0.896
Heart Failure (%)	152 (12.8)	101 (11.6)	51 (16.0)	0.043
CAD (%)	204 (17.1)	137 (15.7)	67 (21.1)	0.030
COPD (%)	198 (16.7)	142 (16.3)	56 (17.7)	0.572
CKD (%)	110 (9.3)	72 (8.3)	38 (11.9)	0.052
Metastatic Cancer (%)	58 (4,9)	30 (3.4)	28 (8.8)	< 0.001
Diabetes Mellites (%)	310 (26.1)	190 (21.8)	120 (37.7)	< 0.001
Charlson Comorbidity Index (IQR)	3 (1 - 5)	3 (1 - 5)	4 (3 - 6)	< 0.001
**Category of Admission**				
**Medical** (%)	652 (54.8)	439 (50.3)	213 (67.0)	< 0.001
**Surgical**				
Elective Surgery (%)	153 (12.9)	132 (15.1)	21 (6.6)	< 0.001
Emergent Surgery (%)	293 (24.6)	230 (26.4)	63 (19.8)	0.020
**Trauma** (%)	157 (13.2)	127 (14.6)	30 (9.4)	0.021
Neurological/ Neurosurgical (%)	274 (23.0)	183 (21.0)	91 (28.6)	0.006
**Patients' origin**				
Ward / Other ICU (%)	459 (38.6)	303 (34.7)	156 (49.1)	< 0.001
ED / Other hospital (%)	379 (31.8)	282 (32.3)	97 (30.5)	0.547
Operating Room (%)	352 (29.6)	287 (32.9)	65 (20.4)	< 0.001
Pre -ICU in-hospital days (SD)	6.16 (16.21)	4.48 (9.91)	10.76 (26.21)	< 0.001
**Patients' clinical condition at ICU admission**				
Respiratory Failure (%)	309 (26.0)	213 (24.4)	96 (30.2)	0.045
Sepsis (%)	173 (14.5)	109 (12.5)	64 (20.1)	<0.001
SAPS II (IQR)	38 (27 - 49)	36 (26 - 45)	47 (37 - 58)	< 0.001
SAPS III (SD)	71.19 (13.52)	67.70 (12.24)	80.76 (12.19)	< 0.001
SOFA (IQR)	8 (6 - 10)	8 (6-10)	10 (8 - 11)	< 0.001
qSOFA (SD)	1.76 (0.81)	1.69 (0.79)	1.96 (0.81)	< 0.001
APACHE II (IQR)	17 (12 - 23)	16 (11- 21)	22 (17 - 28)	< 0.001
APACHE IV (IQR)	56 (41 - 72)	52 (39 - 67)	70 (56 - 88)	< 0.001
Lactate (mg/dl) (IQR)	18.6 (12.0 – 33.0)	18.0 (12.0 – 32.9)	19.0 (12.0 – 34.7)	0.137
GFR (ml/min/1,73m^2) (IQR)	66.1 (41.6 – 90.5)	69.7 (44.4 – 94.0)	54.2 (35.5 – 79.0)	< 0.001
**Data from the Patient's ICU Stay**				
ICU LOS (IQR)	7 (4 - 15)	7 (4 - 14)	13 (6 - 21)	< 0.001
Duration of MV (h) (IQR)	74 (18 - 237)	72 (20 - 207)	199 (61 - 367)	< 0.001
Duration of vasopressors administration (24h) (IQR)	4 (2 - 8)	4 (2 - 7)	7 (3 - 13)	< 0.001
Lactate Clearance 24h (IQR)	-0.34 (-0.56 – 0.0)	-0.33 (-0.56 – 0.0)	-0.27 (-0.52 – 0.1)	0.109
Lactate Clearance 48h (IQR)	-0.40 (-0.66 – 0.1)	-0.42 (-0.67 – 0.1)	-0.37 (-0.63 – 0.2)	0.017
Transfusion (%)	469 (39.4)	283 (32.5)	186 (58.5)	< 0.001
Number of Blood products (IQR)	0 (0 - 2)	0 (0 - 1)	1 (0 - 3)	< 0.001
Infection in ICU (%)	360 (30.3)	226 (25.9)	134 (42.1)	< 0.001
VAP in ICU (%)	162 (13.6)	70 (8.0)	92 (28.9)	< 0.001
CRBSI in ICU (%)	132 (11.1)	44 (5.0)	88 (27.7)	< 0.001
AKI (%)	398 (33.4)	231 (26.5)	167 (52.5)	< 0.001
CRRT (%)	217 (18.2)	118 (13.5)	99 (31.1)	< 0.001
Duration of CRRT (h) (SD)	28.31 (96.81)	16.83 (66.41)	59.70 (147.17)	< 0.001
Delirium (%)	356 (29.9)	273 (31.3)	83 (26.1)	0.083

Total Parenteral Nutrition (%)	202 (17.0)	114 (13.1)	88 (27.7)	< 0.001
**Patients' Clinical Condition at ICU discharge**				
Holidays / Out of hours (%)	214 (18.0)	165 (18.9)	49 (15.4)	0.163
Tracheostomy (%)	336 (28.2)	161 (18.5)	175 (55.0)	< 0.001
Mechanical Ventilation (%)	50 (4.2)	31(3.6)	19 (6.0)	0.065
GCS (SD)	13.67 (2.94)	14.27 (2.12)	12.03 (4.05)	< 0.001
SAPS II (IQR)	24 (16 - 31)	21 (15 0 28)	32 (26 - 40)	< 0.001
APACHE II (IQR)	11 (7 - 16)	10 (6 - 14)	15 (12 - 20)	< 0.001
SOFA (IQR)	3 (2 - 5)	3 (2 - 4)	5 (3 - 6)	< 0.001
qSOFA (SD)	1.07 (0.78)	0.97 (0.76)	1.33 (0.78)	< 0.001
GFR (ml/min/1.73m^2) (IQR)	83.7 (52.4 – 115.5)	87.8 (56.4 – 117.4)	71.6 (46.1 – 112.8)	0.012
WBC (K/μl) (IQR)	11.0 (8.3 – 14.3)	10.7 (8.2 – 13.6)	12.4 (9.1 – 15.4)	<0.001
HgB (g/dl) (IQR)	9.3 (8.5 – 14.3)	9.5 (8.5 – 10.9)	8.9 (8.3 – 9.7)	< 0.001
Lactate (mg/dl) (IQR)	9.6 (7 – 13)	9 (7 - 12)	11 (8 - 15)	< 0.001
CRP (mg/dl) (IQR)	9.44 (3.23 – 14.03)	7.7 (3.3 – 14.3)	6.8 (3.2 – 13.2)	0.176
PaO_2/FiO_2 ratio (IQR)	247 (184.5 – 317.3)	246 (183 - 313)	256 (192 - 332)	0.159

Table S8: Net benefit of the predictive model compared with treat-all and treat-none strategies across selected threshold probabilities.
At clinically relevant thresholds (0.1–0.9), the model consistently demonstrates higher net benefit than a "treat-all" or "treat-none" strategy, particularly between 0.10 and 0.30.

Threshold probability	Net benefit (Model)	Net benefit (Treat all)	Net benefit (Treat none)
0.1	0.207	0.187	0.000
0.2	0.168	0.085	0.000
0.3	0.142	-0.046	0.000
0.4	0.107	-0.220	0.000
0.5	0.088	-0.464	0.000
0.6	0.061	-0.830	0.000
0.7	0.040	-1.440	0.000
0.8	0.016	-2.660	0.000
0.9	0.007	-6.319	0.000

Table S9. Comparison of Baseline Characteristics Between Derivation and Validation Cohorts
Comparison of baseline characteristics between the derivation and validation cohorts. p-values were calculated using appropriate statistical tests (t-test for normally distributed continuous variables, Mann–Whitney U test for non-normal continuous variables, and chi-square test for categorical variables). No statistically significant differences were observed between cohorts, supporting the adequacy of the external validation cohort for the transportability of the WOScore.

Characteristics	Derivation Cohort (n=1190)	Validation Cohort (n=183)	p-value
Age (median, IQR)	64 (50-74)	66 (52-79)	0.20
Male gender (%)	757/1190 (63.6)	109/183 (59.56)	0.29
Diabetes Mellitus (%)	310/1190 (26.1)	53/183 (28.9)	0.26

CCI (median, IQR)	3 (1-5)	3 (0-5)	0.98
Medical Admission (%)	652/1190 (54.79)	96/183 (52.46)	0.56
SAPS III (Admission) (mean +/-SD)	71.19 (13.52)	68.9 (15.54)	0.06
ICU LOS (median, IQR)	7 (4-15)	6 (3-12)	0.068
Tracheostomy (%)	336/1190 (28.24)	40/183 (21.86)	0.072
SAPS II (median, IQR)	24 (16-31)	22 (16-28)	0.31
ICU Readmission (%)	126/1190 (10.58)	21/183 (11.47)	0.72
In-hospital Death (%)	192/1190 (16.1)	21/183 (11.47)	0.11
Worse Outcome (%)	318/1190 (26.72)	42/183 (22.95)	0.28

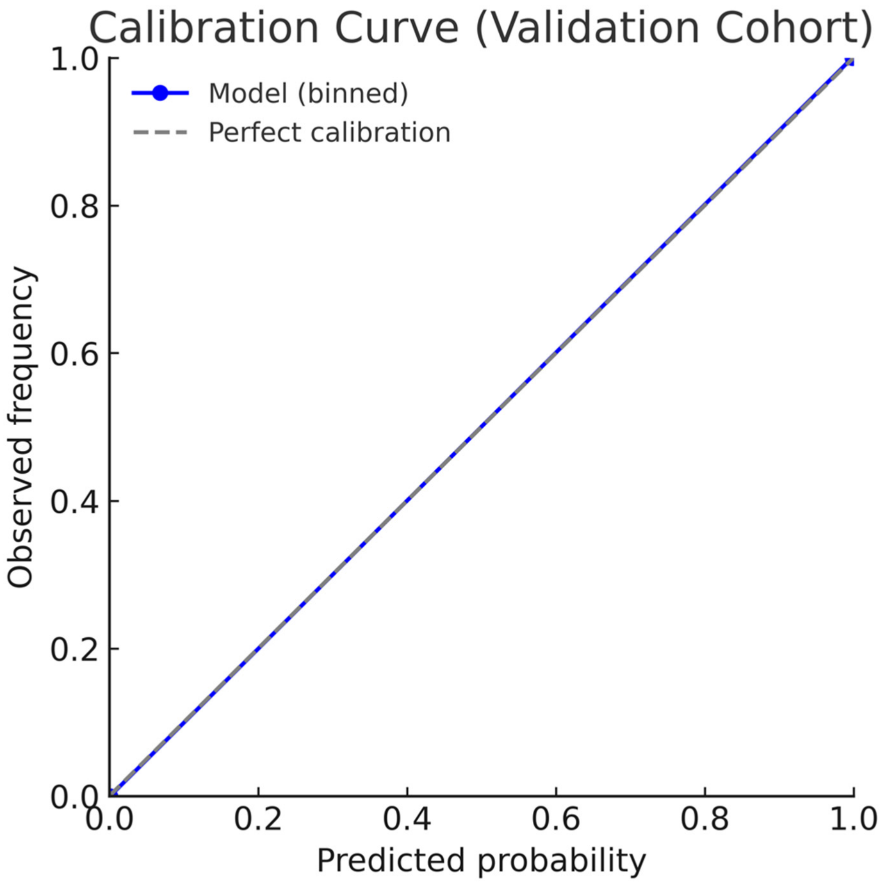

Figure S4: Calibration curve (validation cohort).
Observed event frequencies are plotted against predicted probabilities in deciles of risk (blue line with circles). The dashed gray line represents perfect calibration. The close alignment of the model's predictions with the reference line indicates excellent agreement between predicted and observed outcomes.

Table S10. Net benefit of the predictive model across selected threshold probabilities in the validation cohort.
The table reports the net benefit of the model compared with the "treat-all" and "treat-none" strategies at thresholds ranging from 0.10 to 0.90.

Threshold probability	Net benefit (Model)	Net benefit (Treat all)	Net benefit (Treat none)
**0.1**	0.4	0.33	0.0
**0.2**	0.4	0.25	0.0
**0.3**	0.4	0.14	0.0
**0.4**	0.4	-0.0	0.0
**0.5**	0.4	-0.2	0.0
**0.6**	0.4	-0.5	0.0
**0.7**	0.4	-1.0	0.0
**0.8**	0.4	-2.01	0.0
**0.9**	0.4	-5.01	0.0
